# Optimizing sequencing protocols for leaderboard metagenomics by combining long and short reads

**DOI:** 10.1186/s13059-019-1834-9

**Published:** 2019-10-31

**Authors:** Jon G. Sanders, Sergey Nurk, Rodolfo A. Salido, Jeremiah Minich, Zhenjiang Z. Xu, Qiyun Zhu, Cameron Martino, Marcus Fedarko, Timothy D. Arthur, Feng Chen, Brigid S. Boland, Greg C. Humphrey, Caitriona Brennan, Karenina Sanders, James Gaffney, Kristen Jepsen, Mahdieh Khosroheidari, Cliff Green, Marlon Liyanage, Jason W. Dang, Vanessa V. Phelan, Robert A. Quinn, Anton Bankevich, John T. Chang, Tariq M. Rana, Douglas J. Conrad, William J. Sandborn, Larry Smarr, Pieter C. Dorrestein, Pavel A. Pevzner, Rob Knight

**Affiliations:** 1 Department of Pediatrics, University of California San Diego School of Medicine, La Jolla, CA 92093 USA; 2 Center for Algorithmic Biotechnology, Institute for Translational Biomedicine, St. Petersburg State University, St. Petersburg, Russia; 3 Bioinformatics and Systems Biology Program, University of California San Diego, La Jolla, CA USA; 4 Department of Computer Science and Engineering, University of California San Diego, La Jolla, CA USA; 5 Illumina, Inc., La Jolla, CA USA; 6 Division of Gastroenterology, Department of Medicine, University of California San Diego, La Jolla, CA USA; 7 Inflammatory Bowel Disease Center, University of California San Diego, La Jolla, CA USA; 8 Institute for Genomic Medicine, University of California San Diego, La Jolla, CA USA; 9 Skaggs School of Pharmacy and Pharmaceutical Sciences, University of California San Diego, La Jolla, CA USA; 10 Division of Pulmonary, Critical Care and Sleep Medicine, Department of Medicine, University of California San Diego, 9500 Gilman Drive, La Jolla, CA 92093 USA; 11 Center for Microbiome Innovation, Jacobs School of Engineering, University of California San Diego, La Jolla, CA USA; 12 California Institute for Telecommunications and Information Technology, University of California San Diego, La Jolla, CA USA; 13 Department of Bioengineering, University of California San Diego, La Jolla, CA USA; 14 Skaggs School of Pharmacy and Pharmaceutical Sciences, University of Colorado, Aurora, CO USA; 15 Department of Biochemistry and Molecular Biology, Michigan State University, East Lansing, MI USA; 16 Collaborative Mass Spectrometry Innovation Center, Skaggs School of Pharmacy and Pharmaceutical Sciences, University of California San Diego, La Jolla, CA USA

**Keywords:** Leaderboard metagenome, Long reads, Benchmark, Assembly, Binning

## Abstract

As metagenomic studies move to increasing numbers of samples, communities like the human gut may benefit more from the assembly of abundant microbes in many samples, rather than the exhaustive assembly of fewer samples. We term this approach leaderboard metagenome sequencing. To explore protocol optimization for leaderboard metagenomics in real samples, we introduce a benchmark of library prep and sequencing using internal references generated by synthetic long-read technology, allowing us to evaluate high-throughput library preparation methods against gold-standard reference genomes derived from the samples themselves. We introduce a low-cost protocol for high-throughput library preparation and sequencing.

## Introduction

DNA sequencing of microbial samples has emerged as a technology of choice for analyzing complex bacterial communities. In the past years, the field of metagenomics has been shifting from marker gene-based approaches toward de novo assemblies of shotgun metagenomic sequencing data, followed by binning the resulting contigs into clusters representing individual organisms [1–3]. However, despite many efforts, de novo metagenomic assembly remains challenging. The complexity of many metagenomic samples, combined with widely varying abundance of the constituent species, demands sequencing effort that dwarfs most other applications of next-generation sequencing. This challenge is further amplified in emerging high-throughput projects aimed at sequencing thousands of microbiomes—especially the human gut.

Unfortunately, most individual genomes resulting from metagenome sequencing are often far from the quality standards achieved in assembling bacterial isolates. The first issue is that even with deep sequencing, the coverage of most species is still less than the typical coverage depth in isolate sequencing projects. The second issue is that conserved genomic fragments present in multiple microbial species lead to hard-to-resolve inter-genomic repeats during the assembly process. Finally, the high microdiversity of many bacterial communities leads to additional deterioration of assemblies [4, 5]. These challenges make it impossible to generate high-quality assemblies of individual genomes within a metagenome for all but a few abundant species.

However, metagenomic studies have been rapidly progressing from analyzing a few samples to analyzing many samples. Analysis of multiple bacterial communities of similar origins (e.g., human stool) has revealed that they widely differ in composition [6, 7]. Moreover, analysis of a single community across multiple time points, even in the absence of apparent variation in external conditions [8–11], shows rapid and drastic shifts in the community composition. This observation suggests an alternative sequencing strategy that focuses on analyzing abundant species in multiple datasets rather than increasing the sequencing depth and sensitivity of the analysis of a single sample. This strategy, which we refer to as *leaderboard metagenomics*, is also supported by the recent success of binning algorithms based on differential coverage of genomic fragments across multiple samples [2, 12, 13]. The resulting set of leaderboard genomes can then be used for mapping-based analysis of less abundant species and strain variants within each sample. The leaderboard approach to metagenomic assembly is implicit in the use of co-abundant gene groups to partition metagenomes [3], and tools for dereplicating redundant genome bins from individually assembled samples [14] have been used successfully in meta-analyses of publicly available metagenomic data to dramatically increase the breadth of the available human-associated microbial genome catalog [15].

While the increased sample size has clear theoretical advantages, most research is resource-constrained, and individual investigators have to weigh the benefits of a higher sample size with the costs of generating additional sequencing libraries. Current sequencing protocols have significant performance differences in metagenome studies [16]. To scale leaderboard metagenomics to thousands of samples and to maximize its efficiency, it is imperative to benchmark experimental approaches both in terms of cost and assembly quality. While the quality of genome assemblies is usually assessed on isolates with known reference genomes [18, 19], benchmarking of metagenome assemblies is a more difficult task because reference metagenomes are rarely available. This problem is typically addressed by generating synthetic mock datasets with known community members [20–22].

In this work, we propose a different path for benchmarking metagenome assemblies which uses synthetic long-read sequences as a reference. Using long reads permits benchmarking protocols directly on the community of interest without having to assemble mock samples, while simultaneously generating a complementary sequence that can be used for improved hybrid assembly. Since TrueSeq synthetic long read (TSLR) technology [23, 24] yields high-quality reconstruction of abundant microbial species [25, 26], it is ideal for benchmarking leaderboard metagenomic protocols, although the same concepts apply to other highly accurate long-read technologies as they emerge [27–29]. We exploit tools of SPAdes family [25, 30, 31] to assemble short-read data and TSLR data and use metaQUAST [32] for evaluating the quality of short-read assemblies with the TSLR-derived genomic bins as the underlying references. We benchmarked three sequence library preparation protocols (TruSeqNano, NexteraXT, and KAPA HyperPlus) for performance in leaderboard metagenomics of the human gut microbiome. We then used these data to guide the development of a high-throughput, miniaturized library preparation protocol that dramatically reduces per-sample costs, facilitating the application of a leaderboard metagenomics approach to new datasets. We make these data, as well as the automated workflow for comparative assessment, available as a community resource so that alternative assembly tools and novel metagenomic environments can be easily benchmarked in subsequent works.

## Results

### Sequencing parameter cost/benefit analysis

To ensure that our subsequent comparisons of library preparation protocols were performed using cost-effective sequencing parameters, we did an initial assessment of assembly results given cost-matched sequencing effort on different sequencing instruments. We calculated the per-gigabase sequencing cost using Rapid Run flow cells on Illumina HiSeq2500 and HiSeq4000 instruments at 150 bp and 250 bp paired-end (PE) read lengths. In general, sequencing was most cost-effective using the HiSeq4000 instrument at the 150 bp insert size (Additional file 1: Table S1).

However, a given sequencing depth may still perform differently for assembly depending on the insert size, read length, and instrument used. Thus, we compared assembly performance at different insert sizes given cost-matched sequence efforts for HiSeq2500 and HiSeq4000 sequencers, using eight human fecal metagenomes prepared using the TruSeqNano kit (Additional file 1: Table S2). Given the estimates in Additional file 1: Table S1, 1 million reads of HiSeq2500 PE250 costs about the same as 2.4 million reads of HiSeq4000 PE150. We therefore subsampled these libraries to the maximum number of reads available across parameters combinations, cost-matched for the different sequencer types (4.5 million and 10.9 million reads for HiSeq2500 and HiSeq4000, respectively).

**Fig. 1:**
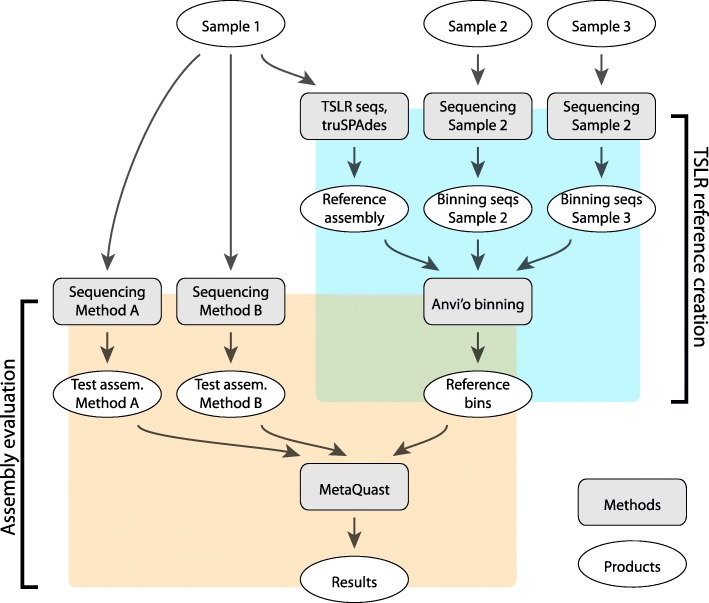
Illustration of the benchmarking workflow using sample 1 as “primary.” Data products are represented by white ellipses and processing methods by gray rounded rectangles. The workflow consists of two parts. In the first part (TSLR reference creation), TSLR data are generated and assembled for primary sample 1. Coverage information from additional samples is used to bin the TSLR contigs into reference genome bins. In the second part (Assembly evaluation), primary sample 1 is sequenced using various short-read sequencing methods. Assemblies from these alternative methods are then compared against the internal reference to benchmark performance

In general, shorter insert sizes yielded superior assemblies in the HiSeq4000 instrument, while longer insert sizes performed better in the HiSeq2500, consistent with the narrower insert size range recommendations from Illumina. Scaffolds of 3 kbp or longer accounted for a median of about 110 total megabases for both HiSeq4000 PE150 libraries using 400-bp inserts and HiSeq2500 PE250 libraries using 1000-bp inserts (Additional file 1: Figure S1). Assembly of very long scaffolds (≥ 50 kbp) was marginally less successful for HiSeq2500 PE250 libraries at these insert sizes, with a total length above this scaffold size at about 92% compared to HiSeq4000 PE150 libraries in matched samples (Fig. 1).

All told, we consistently achieved the best assembly contiguity using HiSeq4000 PE150 sequencing with insert sizes centered around 400 bp; these parameters were used for the remainder of the analyses.

### Creation of internal reference genome bins

We used TruSeq long-read sequencing technology to generate synthetic long-distance reads from eight human fecal microbiome samples, further assembling them into longer contigs per sample (see the “Methods” section). We identified reference genome bins from the TSLR genome assemblies using differential coverage information across samples with the CONCOCT binning algorithm [2] as implemented in the Anvi’o metagenomics pipeline [33], manually refining the bin assignments using the Anvi’o interactive bin refinement tool (Fig. 1) (note that CONCOCT has subsequently been shown to underperform other available binning tools [20]). These refined bins were then scored using a metric incorporating both estimates of genome completeness and purity and average coverage depth in the original sample (see the “Methods” section). For each of the eight samples, we extracted five top-scoring bins for use as internal reference genomes that further served for benchmarking different short-read sequencing strategies. Information resulting in internal references is summarized in Additional file 1: Table S2.

### Assessing assembly quality using reference genome bins

We used the genome bins created above as internal references to evaluate alternative library preparation methods with respect to leaderboard sequencing of human fecal metagenomes. For all eight samples for which we had generated TSLR references, we generated libraries using TruSeqNano and NexteraXT preparation kits and sequenced using a HiSeq4000 sequencer and PE150 sequencing with 400-bp insert sizes. For four of these samples, we also generated libraries using the KAPA HyperPlus preparation kit. A randomly sampled set of ten million read pairs from each of these libraries (the maximum available across libraries) was assembled with metaSPAdes [30] and compared to the reference genome bins using metaQuast [32].

**Fig. 2:**
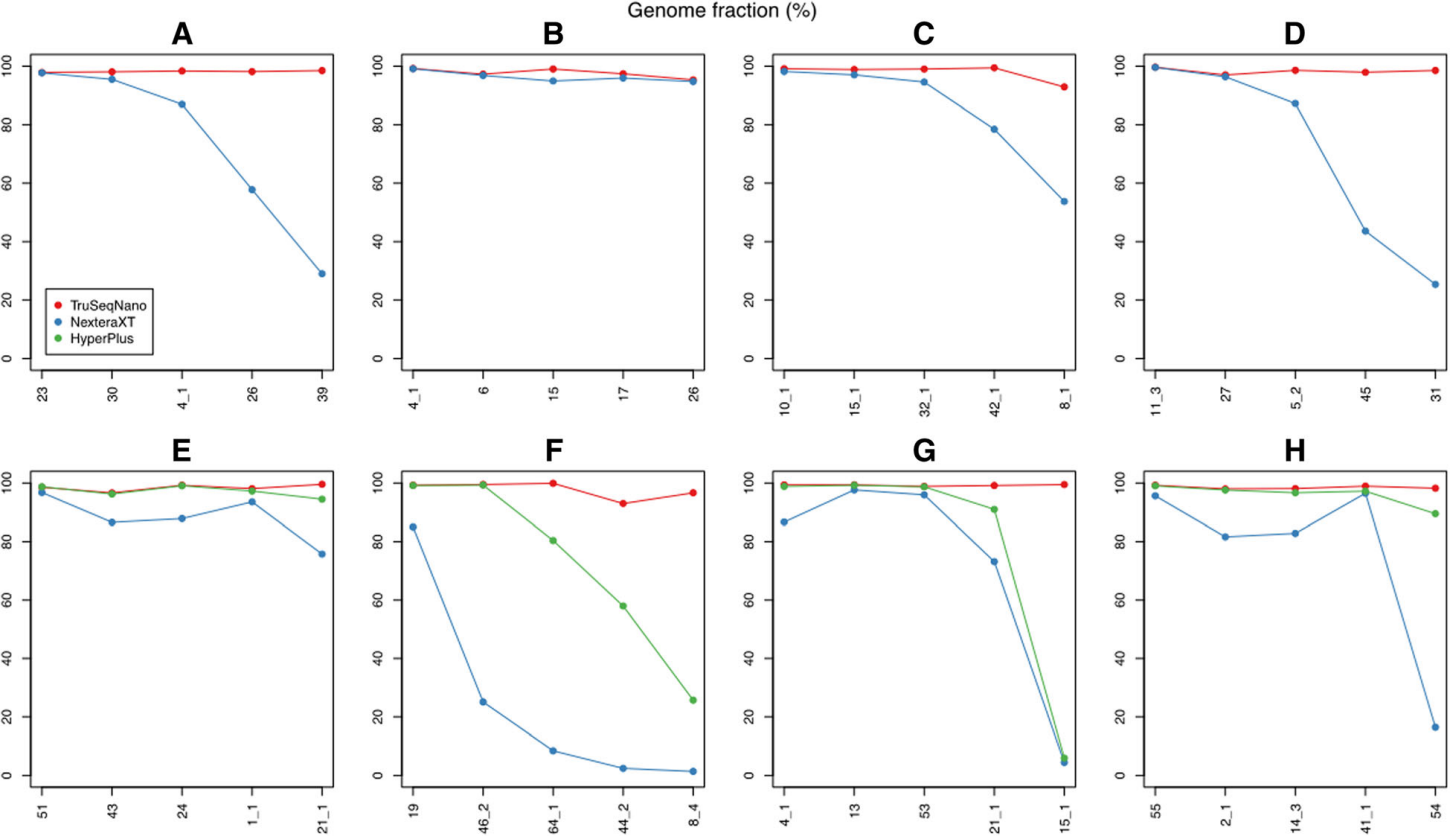
**a**–**h** Genome fraction of internal reference bins recovered in test assemblies. Each panel depicts the performance of the top five reference bins from a separate sample. Reference bins are ordered from the highest to the lowest average recovered genome fraction across the library prep methods tested for that sample (*x*-axis categories are not comparable between panels)

In general, libraries prepared using TruSeqNano technology performed the best with respect to assembled genome fraction, recovering nearly 100% of the 5 reference bins from each of the 8 samples in assemblies (Fig. 2). For NexteraXT libraries, 26 out of 40 total reference genomes were recovered at ≥ 80% completeness (at least 1 bin was recovered at more than 95% completeness in 7 out of the 8 samples). KAPA HyperPlus libraries generally performed better than NexteraXT, with assembly fractions similar to TruSeqNano libraries for 11 of the 20 references in the 4 samples for which data were available (difference < 1%). With respect to per-reference assembled genome fraction (length assembled into contigs ≥ 500 bp), TruSeqNano assemblies were almost strictly better than HyperPlus assemblies, which were in turn strictly better than NexteraXT assemblies.

Per-nucleotide error statistics (mismatches between the assembly and the TSLR reference sequence) were similar among various library preparation methods. This may reflect errors in the underlying TSLR references, or systematic differences in coverage among respective reference genome bins, with lower-abundance genomes having greater proportions of the short-read assembly mapping to low-coverage regions of the TSLR reference with limited capacity for internal error correction (Additional file 1: Figure S2). Although TSLRs feature a lower error rate (below 0.1% on average) than the standard Illumina reads [24], they are not as accurate as the assembled contigs that often have a very small ≈ 0.001% error rate. Additional file 1: Figure S2 illustrates that the mismatch rates for the majority of references were in line with the estimated mismatch rates in TSLRs; 35/40, 27/40, and 17/20 genomes had mismatch rates below 0.1% (1 mismatch per 1000 bp) for TruSeqNano, NexteraXT, and HyperPlus assemblies, respectively. In general, the references with higher assembled genome fractions also had lower mismatch rates. In contrast, indel rates were more systematically different between library prep methods, with NexteraXT libraries having a much higher estimated indel rate than either TruSeqNano or HyperPlus libraries (Additional file 1: Figure S3).

Systematic differences between library prep methods were also quite clear in assembly length statistics, with TruSeqNano libraries almost always having both the longest overall contig (Additional file 1: Figure S4) and the largest fraction of the assembly in contigs greater than 10 kbp (Additional file 1: Figure S5). NexteraXT libraries rarely yielded any contigs greater than 50 kbp in length and typically had very low fractions of the reference genome assembled into ≥ 10 kbp contigs. HyperPlus libraries performed in between on both metrics.

Because we only investigated a single long-read technology as a reference, we cannot eliminate the possibility that differences in performance are in part due to similarities between the TSLR chemistry and short-read chemistries, rather than differences in overall assembly performance. However, the differences we observed in reference assembly statistics mirror differences we observed in non-reference-based statistics—i.e., assemblies were not only more contiguous in comparison with synthetic references, but also using de novo metrics for unrelated samples (see below)—suggesting that similarities between long-read and short-read library chemistries are not the sole explanation.

### Ultra high-throughput miniaturized library prep for leaderboard metagenomics

While full-scale TruSeqNano libraries yielded the most complete assemblies according to our TSLR synthetic references, the labor- and sample-intensive initial fragmentation step makes it relatively difficult to implement at large scale. Methods using enzymatic fragmentation, including NexteraXT, are more amenable to scaling and miniaturization [34]. Given that our evaluation showed that the HyperPlus chemistry (which also uses enzymatic fragmentation) resulted in improved assemblies over NexteraXT at full scale, we implemented a miniaturized, high-throughput version of the HyperPlus library protocol (Additional file 1: Figure S6). We compared its performance to both full-scale libraries using synthetic references and to an implementation of a miniaturized NexteraXT protocol using a panel of real samples.

The miniaturized HyperPlus protocol uses automated acoustic liquid handlers, allowing a 10-fold reduction in reagent volumes in addition to a substantial reduction in consumable pipette tips. It also implements the iTru adapter chemistry [35], which in combination with the acoustic liquid handler allows programmatic addressing of individual wells and thus flexible combinatorial barcoding using 384 unique error-correcting 5′ and 3′ indices. Our implementation of the protocol resulted in a consumable cost of approximately $7 per sample, using manufacturers’ catalog prices, when preparing 384 libraries at a time. Complete overall costs, including capital and operating expenses for liquid handlers, will be higher.

**Fig. 3:**
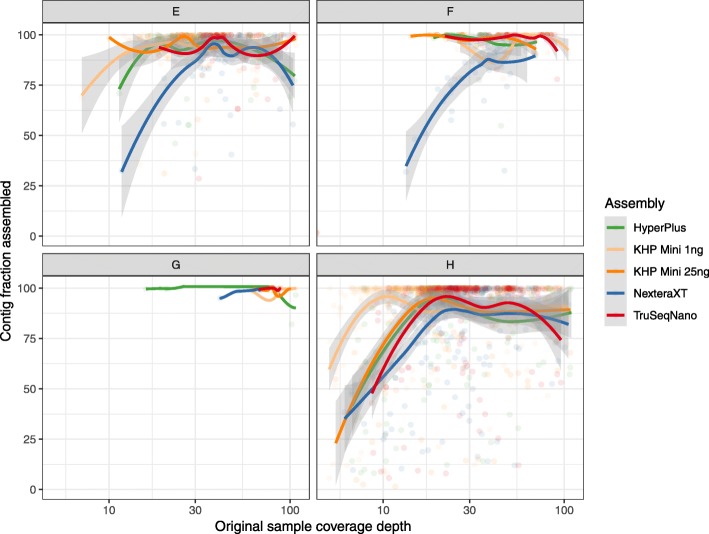
Assembly performance as a function of estimated genome abundance. Points represent the total fraction of a TSLR reference contig assembled as a function of average read depth for that contig, per library prep methodology. Samples **e**–**h** correspond to samples **e**–**h** in Fig. 2

Using TSLR synthetic references for comparison, the miniaturized HyperPlus protocol yielded metagenome assemblies that were comparable to full-scale HyperPlus libraries and superior to full-scale NexteraXT libraries. In particular, we observed improvements in the assembly of lower-coverage portions of the metagenome. To visualize the assembly performance as a function of estimated genome abundance in the original sample, we used individual contigs (rather than bins) from the TSLR assemblies as references, using average read depth from read mapping of the original TruSeqNano libraries as a proxy for genome abundance. In two of the reference samples, NexteraXT libraries showed a decrease in assembly completeness at higher estimated levels of coverage than other chemistries (Fig. 3). This may be due to the localized regions of lower coverage fragmenting assemblies. By comparison, the miniaturized HyperPlus protocol yielded assemblies comparable to TruSeqNano and full-scale HyperPlus protocols across different estimated contig abundances.

We next explored the sensitivity of the protocol to variation in PCR cycle numbers, adaptor concentration, and DNA input. When comparing libraries of the same input biomass, increasing the PCR cycle from 15 to 19 cycles did not change the total number of PCR duplicates (pre-trimming; Additional file 1: Figure S7a) nor the total number of reads (post-trimming; Additional file 1: Figure S7b). The DNA input amount (total mass), however, was negatively associated with PCR duplicate counts, particularly when samples had less than 100 pg. Further, the total read counts was positively associated with DNA input amount (Additional file 1: Figure S7). Based on these results, we chose a standard input DNA amount of 5 ng and 15 PCR cycles. In the same experiment, 2 adaptor concentrations were also tested (360 nl 15 μM vs. 36 nl 15 μM). When less adaptor was added (36 nl 15 μM), PCR duplicates were significantly lower across all 4 DNA input amounts (Additional file 1: Figure S8a, Mann-Whitney). Starting DNA mass was overall negatively correlated to PCR duplicates, with 1 ng (36 nl at 15 μM) input having a median of 1.87% while 1 ng (360 nl at 15 μM) had a median of 15.1%. Furthermore, total read counts were higher for samples processed with the lower adaptor quantities (Additional file 1: Figure S8b). For the final production scale, we dilute primers to 1.5 μM and add 360 nl. In the second experiment, we validated our finalized protocol by sequencing 2 microbial controls across 7 orders of magnitude of input quantity, ranging from 140,000 to 0.14 estimated genome equivalents. Our miniaturized workflow produced libraries with negligible contamination across 4 orders of magnitude of DNA starting material (140,000–140 genomes; 500 pg–500 fg; Additional file 1: Figure S9). The lower limit of detection of this assay was around 500 fg of microbial DNA or approximately 140 genome equivalents.

Next, we performed a direct comparison of miniaturized high-throughput protocols using a panel of samples, including 89 fecal microbiomes from the American Gut Project [36], 84 samples from a time series of human microbiomes from different body sites [8], and 184 bacterial isolates. In addition to the miniaturized HyperPlus protocol, we prepared libraries for all samples using a miniaturized implementation of NexteraXT [37]. We compared assembly performance at shallow depths more commonly used for isolate resequencing (384 samples, including no-template controls, per HiSeq4000 lane; about 0.3 Gbp per sample) and, for metagenomes, at more moderate depths (96 samples per lane; about 1.2 Gbp per sample).

**Fig. 4:**
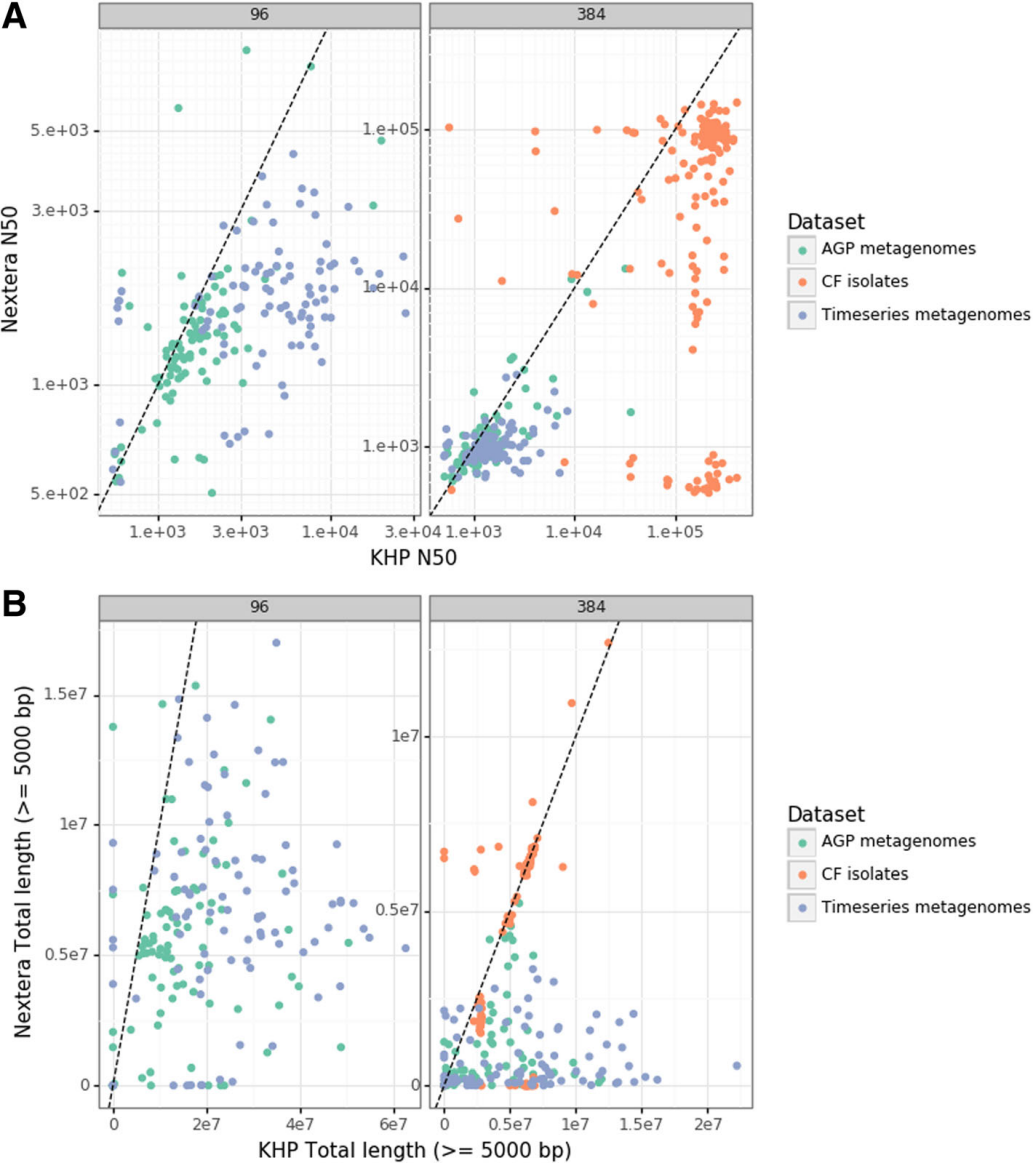
Assembly metrics for miniaturized libraries prepared from three different sample sets. **a** N50 values for samples (points) assembled from miniaturized HyperPlus libraries (horizontal axis) and from miniaturized NexteraXT libraries (vertical axis). Point of equality is indicated by a dotted line, and values are presented for assemblies at a depth of 96 samples per lane (left panel) and at 384 samples per lane (right panel). **b** The total length of assemblies in contigs exceeding 5 kbp in length

Miniaturized HyperPlus libraries generally outperformed miniaturized NexteraXT libraries, especially at more challenging sequencing depths. Most isolates showed similar assembly statistics for each library, indicating that these assemblies were likely limited by genome structure and read length rather than library quality, although a substantial fraction of these samples appeared to fail outright using the NexteraXT chemistry (Fig. 4). For metagenomes, assemblies from miniaturized HyperPlus libraries were almost invariably larger and more contiguous. These differences were least pronounced for metrics like total length (Additional file 1: Figure S10) and most pronounced for metrics emphasizing contiguity, such as the total length assembled in contigs exceeding 50 kbp, where HyperPlus libraries commonly yielded megabases of assembly and NexteraXT almost never yielded any (Additional file 1: Figure S11).

### Leaderboard metagenomics enhances recovery of genome bins

Assembly metrics of our test datasets indicated that, using the miniaturized HyperPlus library protocol, valuable information could be recovered from metagenome assemblies even at sequencing depths much lower than typically performed for complex samples. Given the typical cost of library preparation relative to sequencing, low-coverage metagenome sequencing of large sample numbers is often not cost-effective. However, lower costs and higher throughput afforded by the miniaturized protocol may change this evaluation for some projects.

**Fig. 5:**
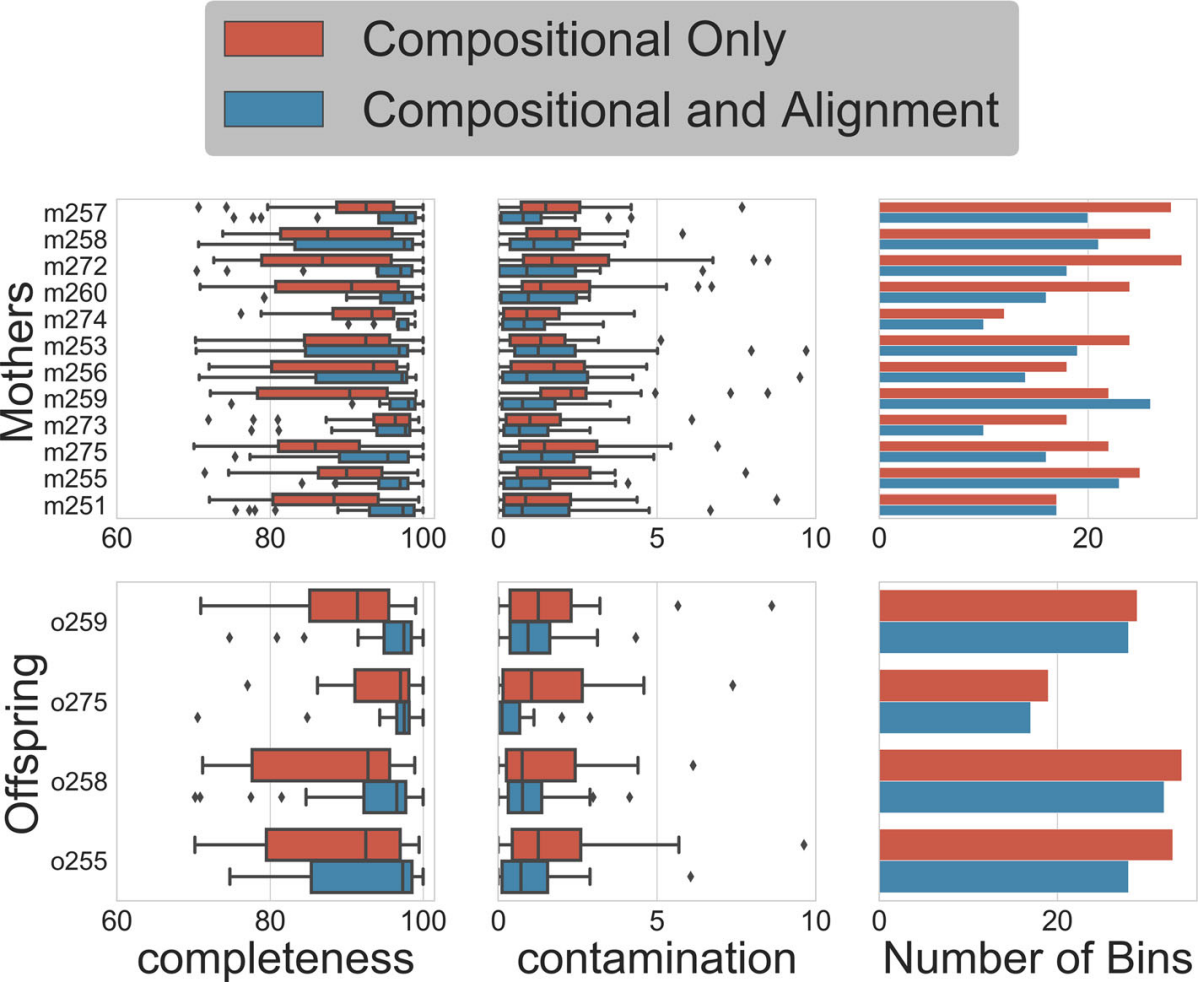
Completeness and contamination statistics for bins recovered from assembly and binning of shallow-sequenced mouse metagenomes. Longitudinal samples for each mother (Mothers) or for each litter (Offspring) were coassembled. “Compositional only” bins were calculated using pooled reads from each longitudinal sample per individual, simulating low-N, high-depth sequencing. “Compositional and alignment” bins were calculated using differential coverage data obtained by mapping each longitudinal sample independently to its individual coassembly

To evaluate the effect of increasing sample number even at lower depths of coverage per sample, we prepared miniaturized HyperPlus sequencing libraries for a set of longitudinal mouse parent/offspring fecal samples. Samples were individually indexed and sequenced at a depth of 384 samples per HiSeq4000 lane. Samples were then co-assembled per individual (mothers) or litter (offspring) and binned using either the per-sample differential coverage and composition information or using pooled coverage and composition information per individual to approximate a lower-throughput but higher-depth sequencing strategy. Incorporating per-time point coverage information improved bin completeness and decreased contamination relative to the pooled time points (Fig. 5). A total of 312 bins exceeding 70% completion and below 10% contamination were recovered, of which 248 exceeded the 90%/5% completeness/contamination thresholds to be considered “high-quality draft” metagenome-assembled genomes [38]. To evaluate the total non-redundant genomic diversity recovered using each method, we dereplicated the total set of genome bins using the dRep pipeline [14]. From the 186 high-quality genome bins recovered using composition-only binning and 248 high-quality bins recovered using per-time point coverage information, we obtained 50 unique genome bins. Of these dereplicated genomes, the highest-quality bin was recovered from the per-time point protocol in 32 cases (Additional file 1: Figure S12).

## Discussion

Long-read internal reference bins enable robust leaderboard benchmarking on real samples, permitting realistic assessment of sequencing and assembly strategies for novel and complex microbial communities. Existing resources for assembly benchmarks, such as in vitro and in silico mock communities [16, 20, 39], have been invaluable for guiding previous sequencing efforts. However, few mock community resources are readily available for other types of environments. Although generating high-fidelity long-read data is also relatively expensive, it does not depend on the isolation and maintenance of organisms, opening benchmarking up to environments where relevant organisms are not present in the culture. And while Illumina has discontinued the TSLR product, other high-fidelity long-read technologies, such as PacBio long-range circular consensus sequencing [27] or emerging tools based on Oxford Nanopore single molecule sequencing [28], should be easily integrated into our approach. Even for technologies which currently lack extremely high fidelity at the single-read level, such as uncorrected single nanopore reads [29], these benchmarks are likely to be especially important as shotgun metagenomic sequencing becomes more widely utilized, and constituent genome assembly challenges become more clinically relevant, since the success of annotating biosynthetic gene clusters and antibiotic resistance cassettes heavily depends on the assembly quality.

In this proof of concept analysis, we have focused on the recovery of genomes present at high abundance, which could be isolated in silico from other genomes in the community. These types of genomes are likely to be central to the expansion of the global microbial genome catalog via metagenome sequencing [12, 15, 40, 41], so assessing the quality of their reconstruction independently of overall metagenomic assembly statistics is an important challenge.

Recent large-scale meta-analyses have dramatically expanded the available genomic catalog for human-associated communities, highlighting the importance of increased sample count to the exploration of global microbial genomic diversity [15, 41]. However, these efforts leveraged the immense investment in human microbiome research over the last decade, analyzing tens to hundreds of thousands of metagenomic samples available in public databases.

For most microbial environments, and most host animal species, such broad-scale community resources do not exist. And while recent research has demonstrated that even shallow metagenomic sequencing can identify the same kinds of biological signals that are recovered from (typically lower-cost) 16S rRNA marker gene sequencing [42], these shallow metagenomic analyses are highly dependent on the quality of the available genome references. We envision leaderboard metagenomics as one way out of this double bind for researchers studying relatively underexplored environments: by trading sequencing depth for increased sample numbers while still assembling the dominant genomes from each sample, we can expand the environment-specific genome catalog organically while improving statistical power to identify biological patterns within individual, moderately scoped studies.

Our results demonstrate that, at least for moderately complex environments like the mammalian gut, shifting effort from increased depth to increased sample number can result in substantial improvements in the quality of genomes recovered from the metagenomic samples. Two important caveats apply. First, the degree of improvement will be a function of the complexity and distribution of microbes across samples. Genomes must be present in multiple samples at varying abundances, and in at least one sample at sufficient abundance for assembly, to benefit. For very complex and evenly distributed communities, like soil microbiomes, there may be few genomes that meet these criteria. Second, microbes can still have important effects at low abundances; even in communities like the mammalian gut, these ubiquitously rare microbes might never be assembled well from more shallowly sequenced samples. In these cases, initial low-coverage sequencing across many samples could still serve to identify targets for higher-depth resequencing efforts. Such a low-coverage high-N approach demands a substantial reduction in the per-sample costs of library construction, while placing a premium on the ability to produce contiguous assemblies at lower average coverage. We found that differences in the library preparation protocol resulted in substantial differences in the assembly of the most abundant organisms and that these differences were exaggerated at lower sequencing depths. Libraries prepared with sonic fragmentation of high input DNA quantities, ligated adapters, and magnetic bead purification are the current standard in the field, and the libraries using this approach in our study (TruSeqNano) were by far the most successful at the reconstruction of underlying internal reference genome bins. However, higher unit cost, labor-intensive fragmentation step, and higher input requirements inhibit the application of these protocols in high-throughput automated pipelines. For these reasons, despite being known to perform poorly in assembly due to unevenness of coverage [43], transposase-mediated protocols such as NexteraXT, which can operate effectively at very low input concentrations and require no separate fragmentation step, have been favored in such applications [44, 45]. Transposase-based libraries have also been implemented in microfluidics-based miniaturization strategies [46, 47].

Our results show that metagenomic libraries generated with the KAPA HyperPlus chemistry, which uses a more automation-friendly enzymatic fragmentation while retaining TruSeq-style adapter ligation, can serve as a useful middle ground. Our miniaturized protocol yields substantial improvements in metagenomic assembly over NexteraXT, while maintaining flexibility in input DNA quantity and reducing consumables costs per sample to a fraction of the per-Gbp cost of Illumina sequencing. By leveraging flexible dual-indexing, it also permits the multiplexing of hundreds to thousands of samples on a single sequencing lane, allowing the cost efficiency of newer NovaSeq sequencers to be accessed even in studies with modest sequencing needs per sample.

Our protocol does rely on automated liquid handling robots to handle reduced fluid volumes and increase throughput. The capital costs associated with the acquisition and upkeep of laboratory robotics, as well as the informatics infrastructure necessary to keep track of tens of thousands of samples and their associated metadata, will limit the number of facilities that will be able to implement it successfully. Other strategies for miniaturization, for example, via purpose-built microfluidics devices, show great promise for reducing the overall capital requirements for high-throughput and low-cost library construction [46, 47].

Advances in throughput and cost-efficiency were critical to the widespread adoption of 16S rRNA gene profiling, and the distributed efforts of researchers across disciplines, study systems, and nations have produced a collective database of marker gene diversity that is beginning to yield insights at a global scale [48]. As surveys of microbial diversity move past the marker gene and toward the metagenome, efforts to increase the utility that each individual study provides to subsequent research can potentially yield enormous dividends—especially for understudied environments and populations. Accurate estimation of genomes from metagenomes is one such dividend: metagenome-assembled genomes can serve both as datasets for testing future hypotheses about genomic content and as references for testing future hypotheses about microbial distribution. By lowering the barriers to sample-specific assembly evaluation and high-sample number metagenome studies, the tools for leaderboard metagenomics we introduce here aim to make genome generation from metagenomes more accessible.

## Methods

### DNA extraction, library preparation, and sequencing

Samples used for the TSLR reference portion of this study were comprised of four human fecal microbiome samples from the Inflammatory Bowel Disease Biobank at UCSD (A-D), as well as four samples spanning approximately yearly intervals from a longitudinal series from a single individual who gave written informed consent (E-H). These studies were both approved by the institutional review board at UC San Diego (IRB protocols #131487 and #14083/#150275, respectively).

Initially, eight libraries were prepared using Illumina TruSeqNano library preparation kits and 100 ng of isolated DNA per sample, and using Illumina NexteraXT preparation kits and 1 ng of DNA, according to the manufacturer’s instructions. Input for TruSeqNano libraries was sheared using a Covaris E220 ultrasonicator. These libraries were purified using AmPure magnetic beads, pooled in equimolar ratios, and different size ranges (< 400, 400–600, 600–800, and 800–1000 bp) selected from purified libraries using a Pippen Prep electrophoresis machine (Sage Sciences). The size-selected libraries were then sequenced on two lanes of a RapidRun-format HiSeq2500 in PE250 mode and on two lanes of a RapidRun-format HiSeq4000 in PE150 mode.

Subsequently, libraries were prepared from four of these samples using a HyperPlus library prep kit (KAPA Biosciences) according to the manufacturer’s instructions. These libraries were prepared with 1 ng of input DNA and 15 cycles of PCR library amplification, pooled, and size selected using the same parameters and instrument as the lowest size range for the above libraries, and sequenced on a HiSeq4000 instrument in PE150 mode.

### TruSeq long-read library preparation, sequencing, and assembly

First, the truSPAdes algorithm [25] was used for the re-assembly of individual synthetic long reads from individual barcoded short-read clouds. Then, the truSPAdes genome assembler [25] was used for the assembly of resulting TSLRs. Normally, SPAdes requires at least one high-coverage paired-end Illumina library for construction of an assembly graph. The truSPAdes algorithm is modified to handle TSLRs as a base for assembly graph construction. In particular, we used iterative assembly graph construction up to a large value of *k* = 127 and, exploiting the high accuracy of the synthetic long reads, introduced a strict threshold on graph processing procedures (such as tip clipper and erroneous connection remover), effectively preventing removal of edges supported by more than two TSLRs. We then extracted contigs from the assembly graph using SPAdes’ hybrid mode [49] designed to use long reads (e.g., SMRT and Sanger) for repeat resolution in the assembly graph.

### TSLR reference bin selection

Assembled TSLR libraries for each of the 8 samples sequenced with TruSeqNano and NexteraXT libraries were processed into contig databases using a Snakemake [50] pipeline adaptation of the recommended workflow for the Anvi’o analysis and visualization platform [33]. This workflow can be found at https://github.com/tanaes/snakemake_anvio. Briefly, contigs for each assembly were indexed and stored in a reference database, then annotated for the presence of several sets of published universal single-copy protein-coding genes [2, 51–53]. Abundance profiles for these contigs were estimated by mapping the reads from each of the eight TruSeqNano libraries to the TSLR assembly using Bowtie2 [54]. Because differential abundance-based binning is most successful with more than 10 samples [2], we also included abundance profiles for the 4 additional samples prepared with HyperPlus library preparation. The abundance profiles from these 12 samples were then used as input to the CONCOCT binning algorithm [2] to group contigs into preliminary bins. The preliminary bins estimated to have high completeness (> 85% universal single-copy marker genes represented) were then manually refined using the anvi-refine tool to reduce the estimated bin contamination (as estimated by the redundancy of single-copy marker genes). Refined bins were scored using the following equation:

$$ {w}_c\times C-{w}_r\times R+{w}_a\times \left(A/{A}_{\mathrm{max}}\right) $$

where *C* is the estimated completeness of the bin (proportion of single-copy genes represented), *R* is the estimated redundancy (based on single-copy genes present in multiple copies), *A* is the abundance of the bin in its original sample as estimated by the average coverage depth, and *A*_max_ is the coverage depth of the most abundant bin in that sample. *w*_*c*_, *w*_*r*_, and *w*_*a*_ are simply the weighting variables chosen to change the contribution of different factors to the score. We used *w*_*c*_ = 1, *w*_*r*_ = 1, and *w*_*a*_ = 10. We chose these values because they yielded bins that were of consistently high quality across these assemblies, enabling automated bin selection in our pipeline.

### Sample assembly and reference comparisons

We constructed an automated pipeline using Snakemake [50] to assemble samples and compare them to the reference bins, available at https://github.com/RNAer/assembly_snakemake_pipeline. The raw sequence reads for each sample were subsampled using seqtk (https://github.com/lh3/seqtk) to an even depth of ten million reads then quality- and adaptor-trimmed using Cutadapt [55]. Subsampled and trimmed paired-end sequences were then de novo assembled using metaSPAdes 3.8.2 [30] using default parameters. Assemblies were then compared against corresponding five highest-scoring internal reference bins from the same sample using MetaQUAST 4. 1[32], which calculates a number of assembly statistics. The taxonomy of each bin was assessed using Taxator-tk 1.3.0e [56] against its reference database “microbial-full_20150430.” Once initiated, the intelligent workflow tracking provided by Snakemake allows test sequences from additional library prep protocols to be sequentially added and compared to prior results, using the minimum necessary re-computation. As new protocols and sequencing technologies become available, this approach will allow analysis teams to maintain an updated evaluation of how different methodologies perform on their unique sample types.

### High-throughput miniaturized HyperPlus library protocol and validation

We developed a high-throughput version of the HyperPlus library chemistry (Kapa Biosciences) miniaturized to an approximately 1:10 reagent volume and optimized for nanoliter-scale liquid-handling robotics. An exhaustive step-by-step protocol and accompanying software are included in Additional file 2. We performed two primary experiments to both optimize and validate miniaturization steps for library preparation. To optimize the PCR cycle number and determine the ideal concentration of barcode adaptors, we choose two high diversity metagenome samples (human feces) and 2 microbial isolates (*Bacillus subtilis* 2610 and *Vibrio fischeri* ES114). Four 10-fold serial dilutions of the samples’ normalized gDNA were performed and used as input for the library preparation representing 1 pg, 10 pg, 100 pg, and 1 ng of gDNA. Sample dilutions were processed in duplicate at 2 adaptor concentrations (15 μM at 360 nl vs. 15 μM at 36 nl). In addition, samples were also processed through either 15 or 19 PCR cycles. The second experiment was conducted in order to validate the final protocol and determine the linear input range of gDNA possibilities along with determining the limit of detection. Genomic DNA from the Zymo Mock community standards, a low diversity community consisting of 10 unique microbes at relatively equal genomic frequencies, and a single microbial isolate, *Vibrio fischeri* ES114 were used as templates. To test the full input range capabilities, we performed 7 10-fold serial dilutions of each sample in duplicate for a total of 28 samples (ranging from 140,000–0.14 genomes) along with four negative controls. gDNA was processed through the 1:10× HyperPlus protocol utilizing 360 nl of 1.5 μM dual index adaptors and a 15 cycle PCR. Samples were then pooled in equal volume and sequenced on a MiSeq 1 × 50 bp kit and then processed through FASTQC [57], Trimmomatic [58], and taxonomy assigned using Kraken/Bracken [59, 60].

Our standard protocol is optimized for an input quantity of 5 ng DNA per reaction. Prior to library preparation, input DNA is transferred to a 384-well plate and quantified using a PicoGreen fluorescence assay (ThermoFisher, Inc). Input DNA is then normalized to 5 ng in a volume of 3.5 μL of molecular-grade water using an Echo 550 acoustic liquid-handling robot (Labcyte, Inc). Enzyme mixes for fragmentation, end repair and A-tailing, ligation, and PCR are prepared and added in approximately 1:10 scale volumes using a Mosquito HT micropipetting robot (TTP Labtech). Fragmentation is performed at 37 °C for 20 min, followed by end-repair and A-tailing at 65 °C for 30 min.

Sequencing adapters and barcode indices are added in two steps, following the iTru adapter protocol [35]. Universal adapter “stub” adapter molecules and ligase mix are first added to the end-repaired DNA using the Mosquito HTS robot and ligation performed at 20 °C for 1 h. Unligated adapters and adapter dimers are then removed using AMPure XP magnetic beads and a BlueCat purification robot (BlueCat Bio). 7.5-μL magnetic bead solution is added to the total adapter-ligated sample volume, washed twice with 70% EtOH, and then resuspended in 7 μL molecular-grade water.

Next, individual i7 and i5 are added to the adapter-ligated samples using the Echo 550 robot. Because this liquid handler individually addresses wells, and we use the full set of 384 unique error-correcting i7 and i5 indices, we are able to generate each plate of 384 libraries without repeating any barcodes, eliminating the problem of sequence misassignment due to barcode swapping [61, 62]. To ensure that libraries generated on different plates can be pooled if necessary, and to safeguard against the possibility of contamination due to sample carryover between runs, we also iterate the assignment of i7 to i5 indices each run, such that each unique i7:i5 index combination is only repeated once every 147,456 libraries. 4.5 μL of eluted bead-washed ligated samples is added to 5.5 μL of PCR master mix and PCR-amplified for 15 cycles. The amplified and indexed libraries are then purified again using magnetic beads and the BlueCat robot, resuspended in 10 μL water, and 9 μL of final purified library transferred to a 384-well plate using the Mosquito HTS liquid-handling robot for library quantitation, sequencing, and storage.

To further validate this protocol against an existing miniaturized library preparation protocol, we generated a sample set comprising 89 fecal microbiomes from the American Gut Project [36], 84 samples from a time series of human microbiomes from different body sites [8], and 184 bacterial isolates of clinical strains derived from cystic fibrosis sputum. The isolates were processed and characterized at the clinical microbiology laboratory in the Center for Advanced Laboratory Medicine (CALM) at UC San Diego. After use for diagnostic purposes, the culture plates were deidentified and collected from CALM. The microbial community was selected from each plate, suspended in LB broth containing 20% glycerol, and frozen at − 80 °C. These pure culture and mixed isolates were then cultured in Todd Hewitt Broth in deep-well 96-well plates at 37 °C prior to DNA extraction. DNA was extracted from samples using the MoBio PowerSoil DNA high-throughput isolation kit per the manufacturer’s recommendations. All 357 DNA samples were combined into a single 384-well source plate and libraries prepared using the above protocol. In addition, we prepared libraries from the same source plate using an implementation of the miniaturized NexteraXT protocol from [37]. Briefly, the NexteraXT protocol was miniaturized at a 1/10 ratio based on the kit’s standard protocol. Genomic DNA was normalized to 1 ng input and went through the recommended tagementation and neutralization protocol. Illumina Nextera indices and NPM were added to the tagmented gDNA at .5 μL and 1.5 μL, respectively. The bead cleanup was omitted to increase efficiency and reduce cost, and the libraries were then normalized at equal volumes, 2 μL per sample. All reagent transfers were performed by the Mosquito HTS liquid-handling robot (TTP Labtech, Inc).

Both sets of libraries were quantified via qPCR and pooled to approximately equal molar fractions using the Echo 550 robot, and the final pools (representing 384 samples each prepared via miniaturized NexteraXT and HyperPlus protocols) were sequenced across 4 lanes of a HiSeq4000 instrument using paired-end 150 bp chemistry.

Demultiplexed sequences were quality filtered and adapter trimmed using Atropos [63], assembled using SPAdes [31] or metaSPAdes [30], and quality metrics summarized using Quast [19] and MultiQC [19, 64], all implemented in a custom Snakemake [50] workflow, available at https://github.com/tanaes/snakemake_assemble.

### Leaderboard metagenomics sequencing and assembly evaluation

To demonstrate the utility of low-coverage whole-metagenome shotgun sequencing for recovering genomes from real-world metagenome samples of moderate complexity, we identified a sample set comprising longitudinal time-series sampling for sequencing with the miniaturized HyperPlus protocol. Studies with a longitudinal sampling component are expected to especially benefit from the reduced per-sample costs of this protocol, as time-series designs can generate large numbers of samples from even modest numbers of subjects, and are consequently often cost-prohibitive to analyze using conventional shotgun metagenomics protocols. The sample set chosen comprises 693 mouse fecal samples collected from 12 mothers over 36 time points and 24 offspring across 11 time points with 4 dropout time points. The treatment groups were split evenly both into mothers and offspring groups with groups of 6 and 12 for mothers and offspring, respectively. Offspring were collectively sampled in 4 litter groups. The pregnant mother mice were sampled every 2 days from an age of 50 to 122 days, and methamphetamine treatment began on day 54. The offsprings were born on day 68 and were sampled every 2 days from 21 days after birth until day 122. The mice were distributed into 4 cages, 2 per treatment group. This study was conducted in accordance with approved protocols by the University of California San Diego. All animal work was approved by the Institutional Review Board at the University of California San Diego and was performed in accordance with the Institutional Animal Care and Use Committee guidelines.

DNA was extracted from these samples using standard Earth Microbiome Project protocols [48], with 10–50 mg of fecal material homogenized and purified with the PowerSoil PowerMag DNA extraction kit (Qiagen, Inc.) and a KingFisher magnetic bead purification robot (ThermoFisher Inc). Libraries were prepared from 5 ng of purified DNA per the above protocol and sequenced across 2 lanes of a HiSeq4000 sequencer (corresponding to 384 samples per lane of sequencing).

Demultiplexed sequences were trimmed using Atropos [63], and paired-end reads were merged with FLASH (v. 1.2.11) [65]. The merged reads along with reads that FLASH was unable to merge were then used to assemble with MetaSPAdes (v. 3.13.0) [30] on *k*-mer lengths of 21, 33, 55, 77, 99, and 127. For assembly, all time point samples from single individuals (mothers) or from single litters (offspring) were combined and coassembled. These coassemblies were then binned using MaxBin2 (v. 2.2.4) [66] and MetaBAT2 (v. 2.12.1) [67], either using contig abundance profiles estimated independently per time point for that individual or (to approximate single-sample deep-sequencing approaches) using a single contig abundance profile calculated with the pooled reads. Abundance profiles were estimated by mapping reads against contigs using BowTie2 (v. 2.2.3) [54] and SAMtools (v. 0.1.19) [68]. MetaBAT2 was run with two parameter profiles, and MaxBin2 was run on default parameters. The first MetaBAT2 parameters were less sensitive with a minimum contig length allowed of 1500 and the other parameters on default. The second had more sensitive parameters with a minimum contig length of 3000, minimum edge score cutoff of 80, and a percentage of good contigs cutoff of 98. The three resulting sets of bins were refined into a single set with metaWRAP (v. 1.1.2) [69]. Quality metrics for the resulting refined bin sets were calculated using CheckM (v. 1.0.13) [70] and compared between abundance profile methodologies described above.

All bins, from both compositional only and compositional and alignment-based binning, were pooled across all subjects. The pooled bin set was dereplicated using dRep (v2.3.2) on default parameters [14]. The resulting dereplicated bin set was filtered for bins considered to be “high-quality draft” metagenome-assembled genomes [38]. The final dereplicated and quality-filtered bin set was then compared for “winning” bin origin of either compositional only or compositional and alignment-based binning.

## Supplementary information

**Additional file 1.** Supplemental figures and tables. Supplemental figures and tables referenced in the text.

**Additional file 2.** Detailed miniaturized library prep protocol. Detailed, step-by-step protocol for miniaturized library preparation.

**Additional file 3.** Review history.

## Data Availability

Sequence data used for miniaturized HyperPlus protocol evaluation, including TSLR reads, are available on the Qiita microbiome data portal under study ID 12615 and mirrored to the EBI ENA under accession ERP116677 [71]. Mouse microbiome sequence data are available on the Qiita microbiome data portal under study ID 10537 and mirrored to the EBI ENA under accession ERP116718 [72].
