# Supplementary material for: Optimizing sequencing protocols for leaderboard metagenomics by combining long and short reads
Source: Genome Biol. 2019 Oct 31;20:226. doi: 10.1186/s13059-019-1834-9 (PMC6822431; doi:10.1186/s13059-019-1834-9)
Supplement: Supplementary file 1 — Additional file 1. Supplemental figures and tables. Supplemental figures and tables referenced in the text. [file 13059_2019_1834_MOESM1_ESM.pdf]

Supplemental materials for manuscript titled:

Optimizing sequencing protocols for leaderboard metagenomics by combining long and short reads

Table S1: Per-unit cost comparison calculation for HiSeq sequencing using prices from the UC San Diego Institute for Genomic Medicine core sequencing facility.

Instrument	Cost (USD)	Read length (bp)	Yield (Gbp)	Unit cost (USD/Gbp)	Reads equiv.*	Coverage equiv.*	50-mer coverage equiv.*
HiSeq4000	2875	150	105	27.38	2.435	1.46	1.215
HiSeq2500	5600	150	120	46.67	1.429	0.857	0.714
HiSeq2500	8000	250	200	40.00	1.000	1.0	1.0

*equiv.: normalized by price, with the HiSeq2500 250 bp protocol as a baseline (so the results are always 1.0 for the HiSeq 2500 protocol).

Table S2. Detailed information of the top five TSLR reference bins per sample, including taxonomic assignment, length and composition statistics, and coverage information.

Sample	Million reads	Bin †	Taxon*	Total length (Mbp)	# contigs	N50 (kbp)	GC (%)	Normalized coverage**	Completeness (%)	Contamination (%)	Score ***
A	40.2	23	*Prevotella*	3.116	11	434.1	45.1	103.19	94.78	4.08	100.7
		39	*Bifidobacterium*	2.172	5	915.8	59.36	34.36	97.03	3.57	96.79
		26	*Roseburia*	2.991	41	148.4	45.25	23.22	98.47	5.66	95.06
		4_1	*Bacteroides*	4.666	39	222.6	41.97	39.94	93.21	4.56	92.52
		30	*Bacteroides*	2.604	5	615.6	44.28	59.73	84.76	3.17	87.38
B	19.5	4_1	*Bacteroides*	4.815	21	556	46.33	242.56	97.3	3.9	100.18

		15	*Akkermansia*	2.889	18	335.8	55.35	25.23	98.51	2.35	96.87
		6	*Bacteroides*	5.08	53	146.9	42.25	69.88	98.69	5.3	95.35
		17	*Parasutterella*	2.748	5	1817.6	48.34	43.44	98.29	5	94.5
		26	*Dialister*	2.234	26	133.6	48.44	34.45	97.84	5.18	93.62
C	30.3	10_1	*Bacteroides*	4.51	16	373	46.63	72.30	95.9	2.76	102.8
		42_1	*Ruminococcus*	2.117	6	635.1	41.34	36.90	98.69	5.23	98.39
		15_1	*Eubacterium*	2.114	7	358.3	44.84	74.93	93.04	5.59	97.46
		32_1	*Eubacterium*	2.346	91	35.9	37.67	22.20	93.88	1.9	94.95
		8_1	*Veillonella*	1.949	43	127.2	38.65	13.10	96.22	5	92.97
D	36.9	11_3	Eubacteriaceae	2	2	1993.7	44.9	206.54	97.7	5.23	96.44
		27	Ruminococcaceae	3.055	40	129.3	55.51	149.33	98.51	5.41	95.97
		45	*Ruminococcus*	1.764	37	96.2	40.84	20.35	95.32	4.38	91.33
		5_2	*Eubacterium*	2.415	70	50.6	41.54	23.68	95.57	6.22	89.81
		31	Firmicutes	2.036	108	23.3	52.97	12.11	87.25	1.36	86.12
E	54.4	13	Enterobacteriaceae	4.637	7	1325.6	48.12	79.44	98.87	3.8	105.08
		21_1	Firmicutes	1.716	65	37.8	61.16	31.59	89.89	1.77	92.1
		15_1	*Akkermansia*	2.464	89	41.5	55.26	9.53	92.11	1.36	91.95
		4_1	*Bacteroides*	3.667	187	24.2	42.07	34.76	89.36	3.82	89.91
		53	*Prevotella*	1.633	36	66	53.53	54.25	85.35	4.44	87.74
F	38.1	14_3	*Alistipes*	2.488	45	83.2	54.18	22.46	97.16	3.68	97.08
		54	*Akkermansia*	2.764	61	61.1	55.25	10.05	96.72	2.35	95.98

		55	Ruminococcaceae	2.673	29	167.1	44.54	20.43	96.88	4.83	95.33
		2_1	*Bacteroides*	4.538	78	95.6	41.98	16.17	96.49	4.38	94.7
		41_1	Firmicutes	1.743	66	36.9	59.8	27.65	90.56	1.68	93.31
G	30.2	24	*Escherichia*	4.505	20	513.4	50.67	93.64	89.3	2.84	96.06
		21_1	*Alistipes*	1.969	73	38.1	54.98	16.10	96.26	3.68	94.23
		43	Firmicutes	2.816	36	118.3	45.66	35.15	88.49	3.86	88.23
		1_1	*Bacteroides*	4.715	45	231.4	42.8	46.42	88.25	4.97	88.04
		51	*Prevotella*	1.889	106	23.8	54.21	97.63	78.46	1.77	86.69
H	58.8	19	*Escherichia*	5.078	9	818.4	50.56	43.55	98.87	3.93	100.36
		46_2	*Akkermansia*	2.649	15	341.9	55.63	20.90	94.91	1.72	95.79
		64_1	*Dialister*	1.676	20	183	45.1	10.76	97.52	4.74	94.13
		44_2	*Ruminococcus*	1.904	80	31.6	41.01	5.23	96.52	5.23	91.94
		8_4	Coriobacteriaceae	1.684	51	54.3	52.74	5.71	94.36	4	91.07

* Taxon: Taxonomic group (genus or above) to which the longest total length of contigs were assigned.

** Normalized coverage: observed coverage / number of input reads * 10 million.

*** Score: Bin score calculated using the equation described in Methods - TSLR reference bin selection.

† Bins named with an underscore were manually refined from unsupervised CONCOCT bins showing higher degrees of contamination.

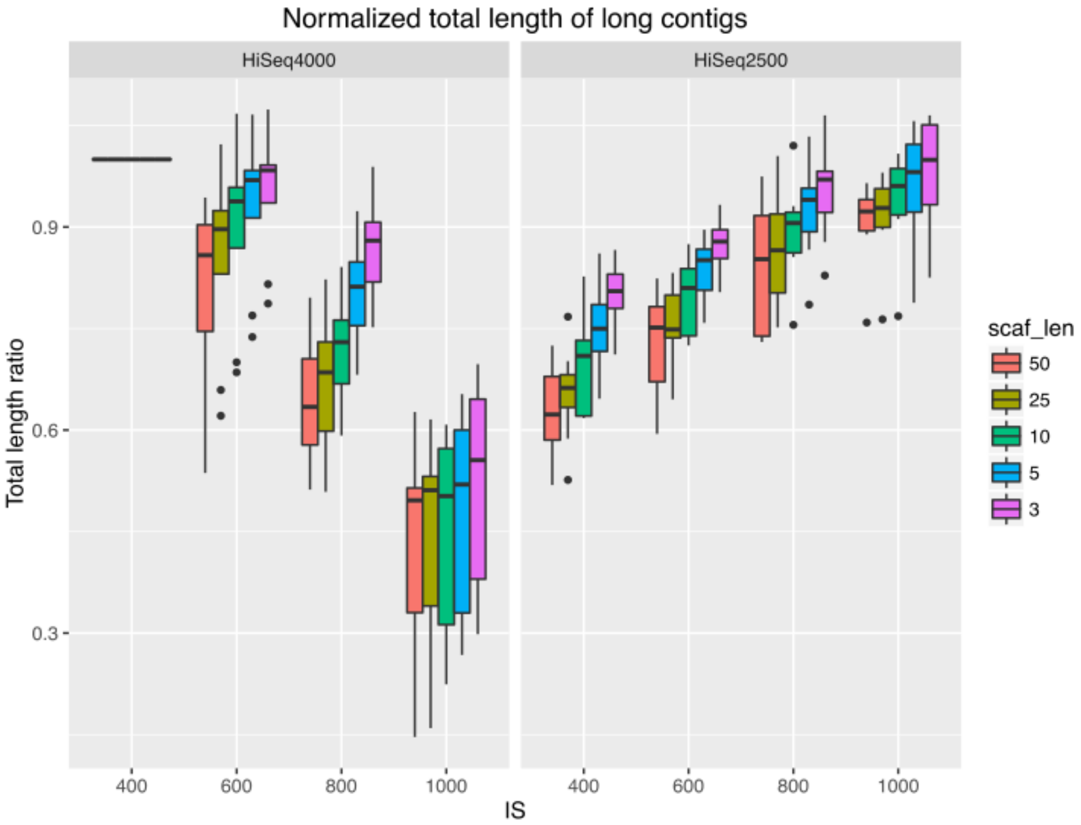

Figure S1. Normalized total length of assemblies of HiSeq4000 PE150 and HiSeq2500 PE250 datasets (after cost-aware subsampling). Total assembly lengths in different scaffold size fractions (≥50, 25, 10, 5, and 3 kbp) are normalized per sample to the value for the corresponding size fraction in the 400bp insert library sequenced on HiSeq4000. Notably, cost-normalized sequencing using HiSeq2500 and the HiSeq4000 yielded dramatically different assembly results for different insert sizes, but with similar overall results for the shorter inserts on HiSeq4000 and the longest inserts on HiSeq2500. This difference in performance is likely driven by a known property of the patterned flow-cell technology in HiSeq4000 instruments to preferentially generate clusters for smaller fragments, which can lead to a bias towards off-target sequences such as adapter dimers when the mean library fragment size is large (http://core-genomics.blogspot.com/2016/01/almost-everything-you-wanted-to-know.html; Fig. S12).

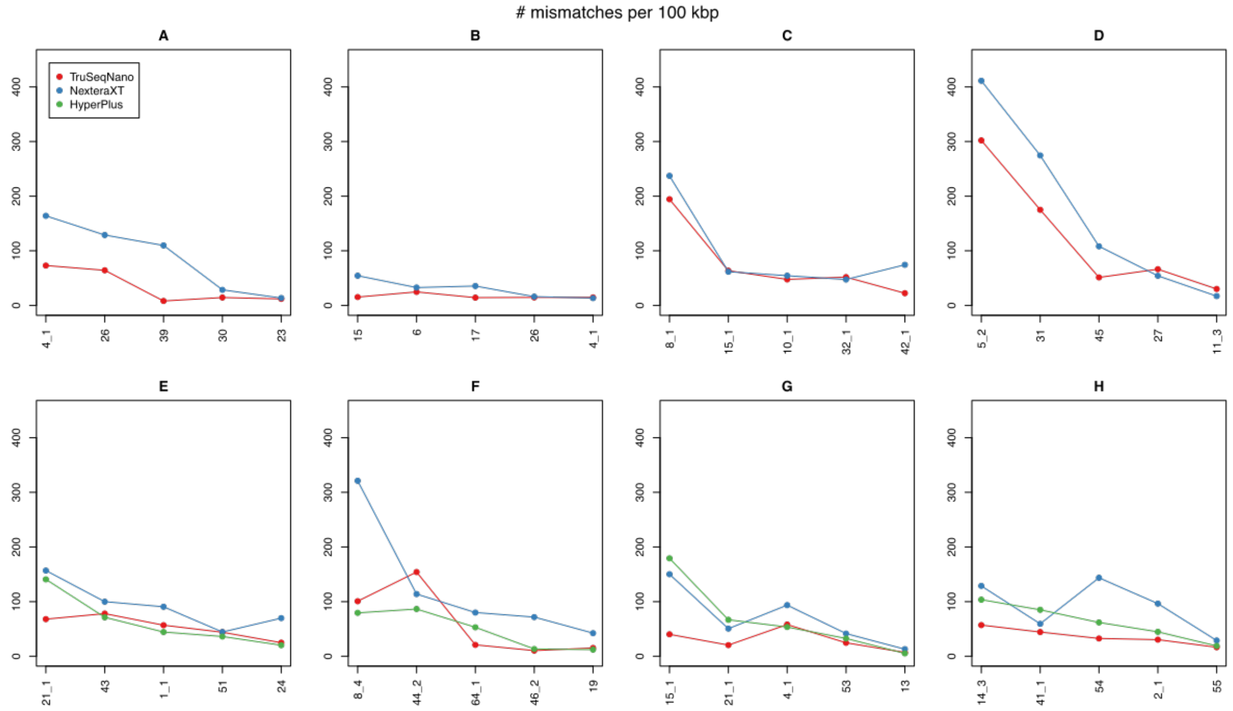

Figure S2. Frequency of mismatches in test assemblies against internal reference bins. Reference bins are ordered from the highest to the lowest number of mismatches per 100 kbp across the library prep methods tested for that sample (x-axis categories are not comparable between panels).

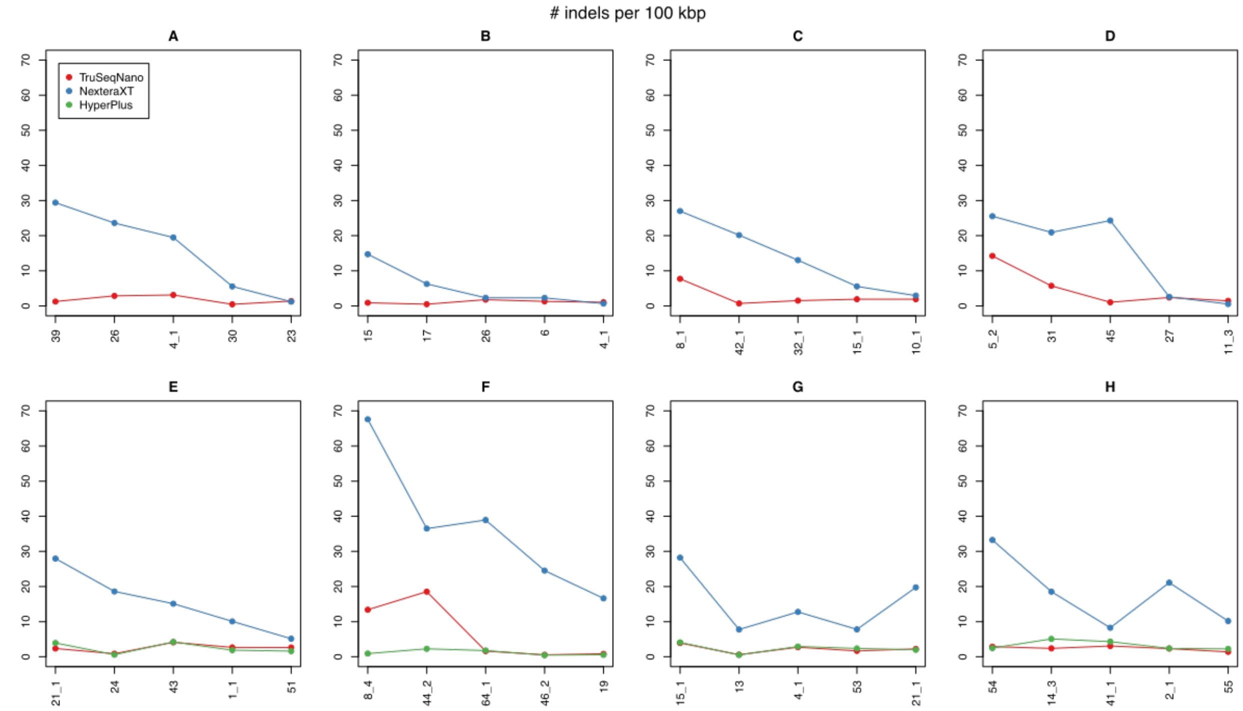

Figure S3. Frequency of indels compared to TSLR reference bins recovered in test assemblies. Reference bins are ordered from the highest to the lowest number of indels per 100 kbp across the library prep methods tested for that sample (*x*-axis categories are not comparable between panels).

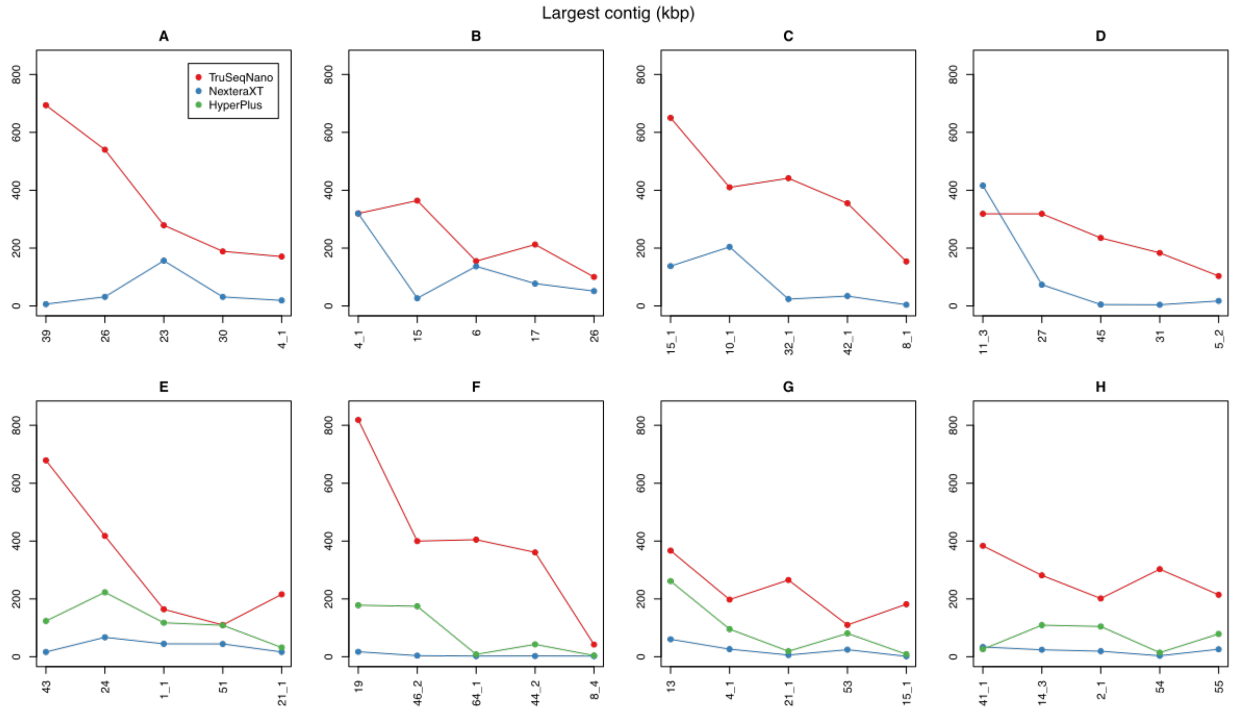

Figure S4. Maximum length (kbp) of contigs mapping to TSLR reference bins from test assemblies. Reference bins are ordered from the highest to the lowest length of the largest contig recovered across the library prep methods tested for that sample (x-axis categories are not comparable between panels).

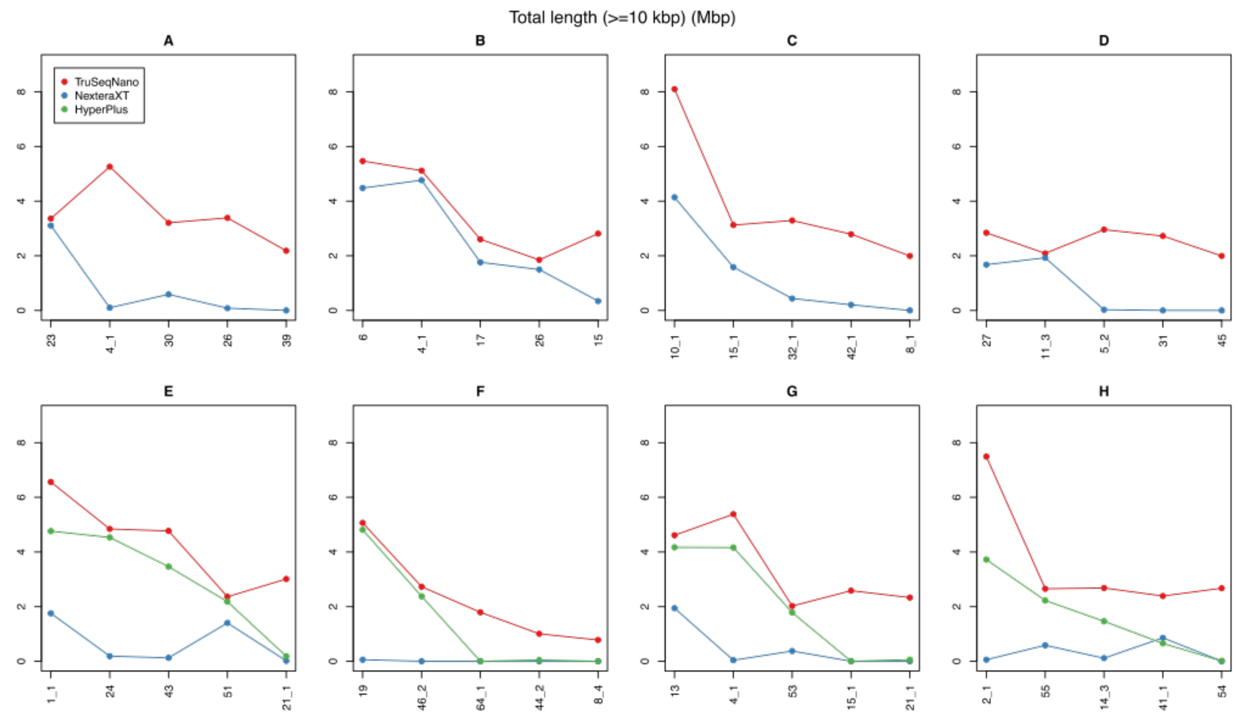

Figure S5. Total length of contigs ≥10 kbp mapping to TSLR reference bins from test assemblies. Reference bins are ordered from the highest to the lowest length recovered in contigs ≥10 kbp across the library prep methods tested for that sample (x-axis categories are not comparable between panels).

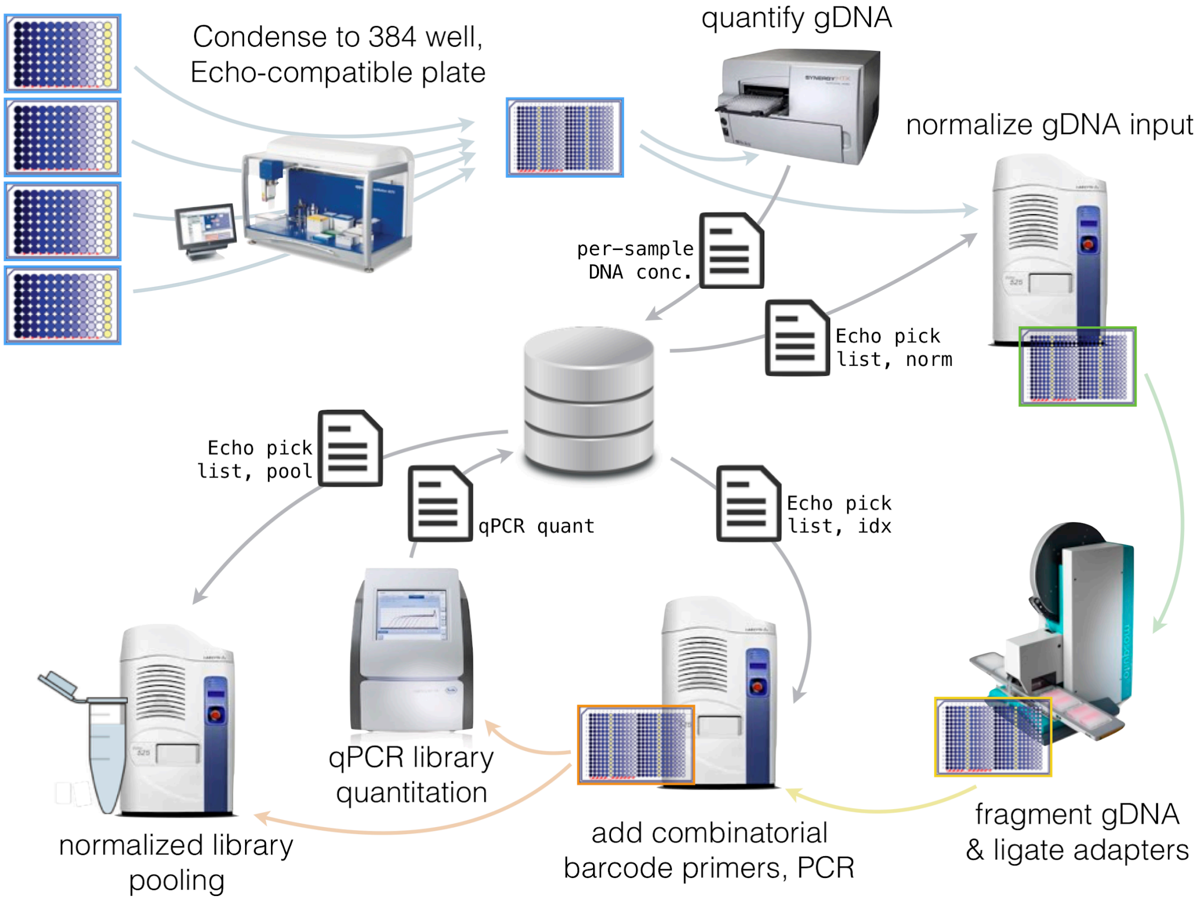

Figure S6. Workflow schematic for miniaturized HyperPlus library construction protocol.

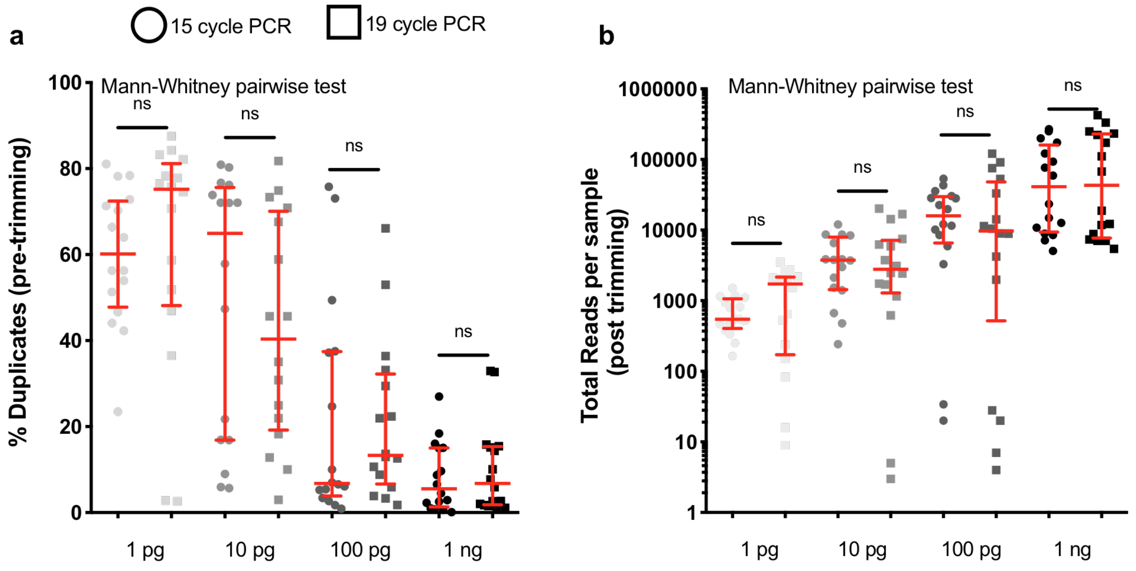

Figure S7. Evaluating effects of PCR cycle number on metagenomic library quality as measured by (a) PCR duplication rate (pre-trimming and QC) and (b) total reads per sample (post-trimming and QC). Distributions are displayed as median, Inter-Quartile-Range. Non-parametric pairwise comparisons (Mann-Whitney) were performed on samples from the same input gDNA biomass.

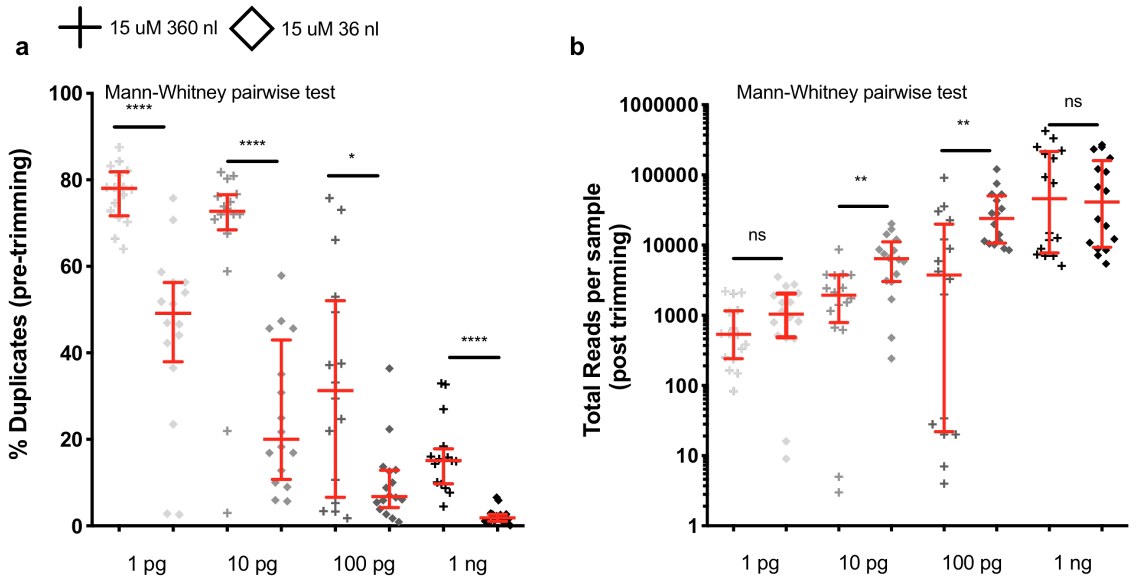

Figure S8. Evaluating effects of adaptor concentration (15 uM at 360 nl vs. 15 uM at 36 nl) on metagenomic library quality as measured by (a) PCR duplication rate (pre-trimming and QC) and (b) total reads per sample (post-trimming and QC). Distributions are displayed as median, Inter-Quartile-Range. Non-parametric pairwise comparisons (Mann-Whitney) were performed on samples from the same input gDNA biomass.

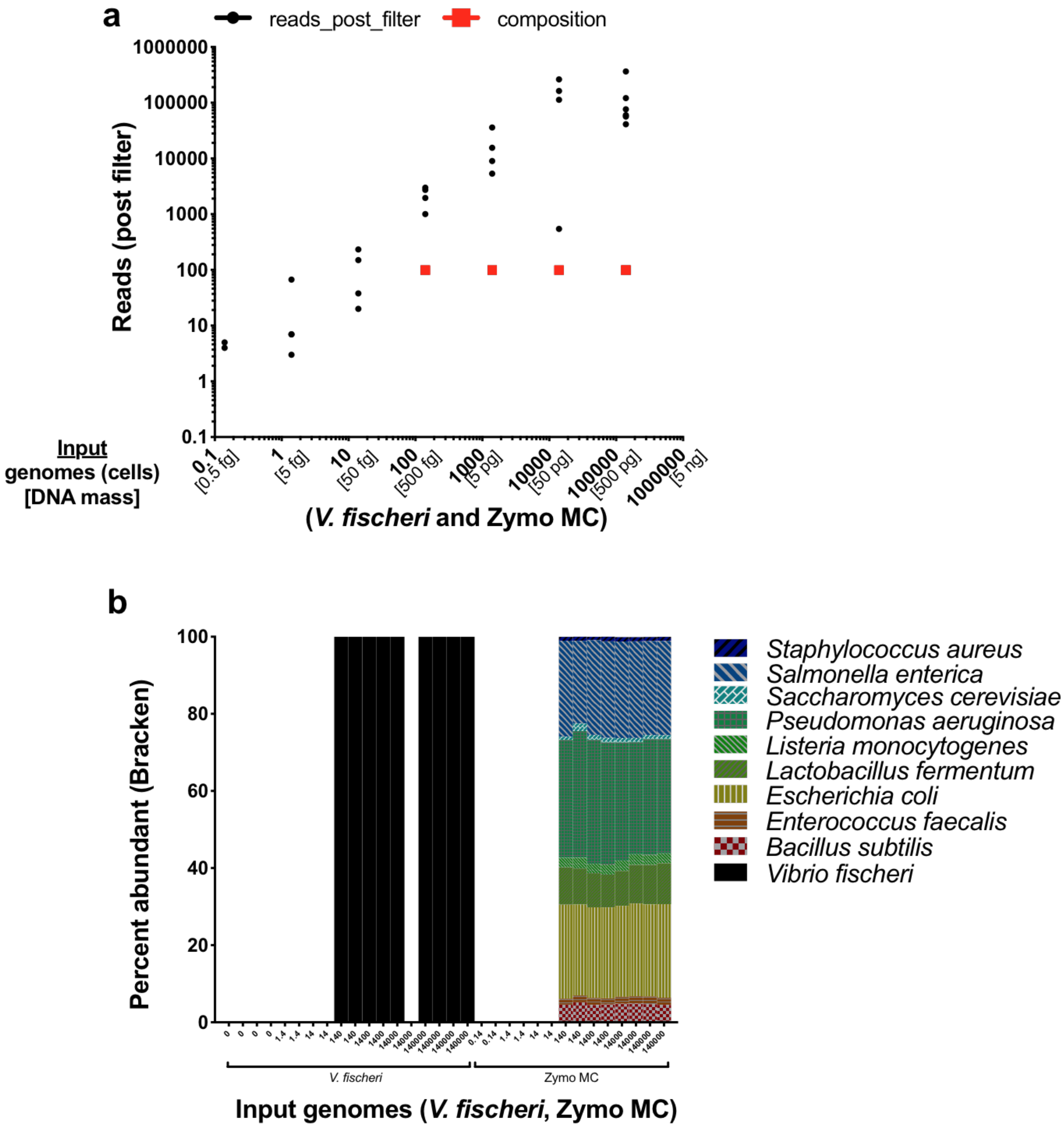

Figure S9. Library preparation input requirements for metagenomic pipeline (final method 360 nl dual index, bluecat cleanup). (a) Linear range, (b) limit of detection to 140 genomes. Samples yielding fewer than 1000 sequence reads after filterning were excluded from the analysis.

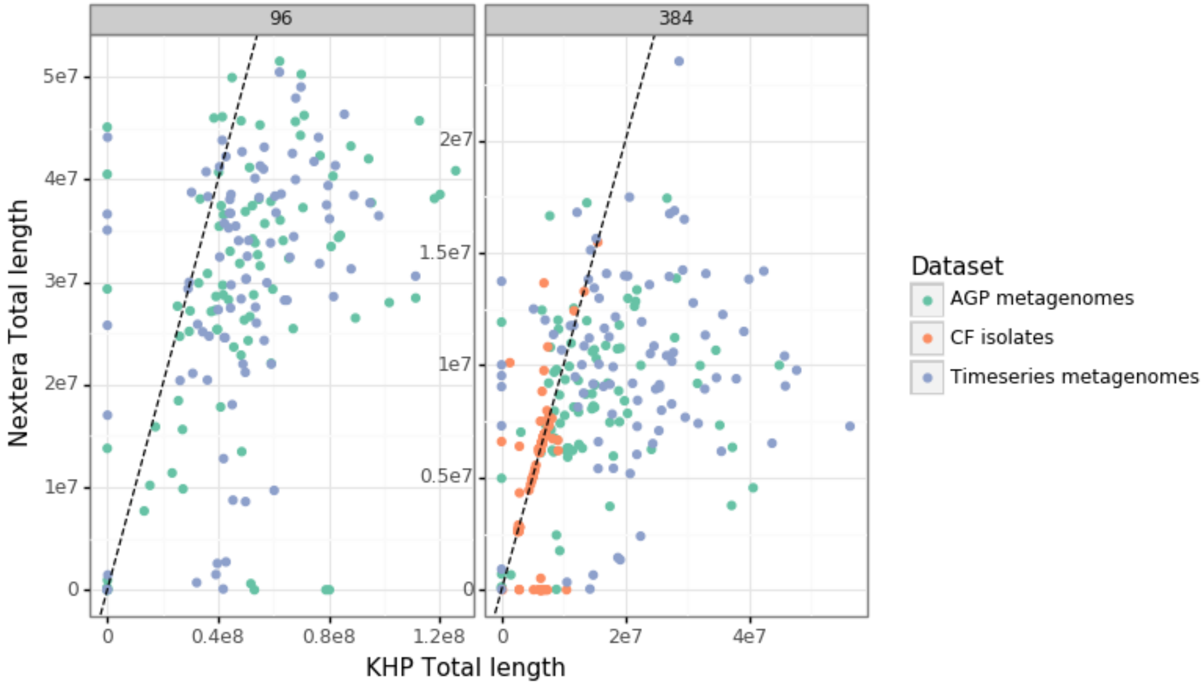

Figure S10. Total length of assembly for miniaturized libraries prepared from three different sample sets. Values for samples (points) assembled from miniaturized HyperPlus libraries (horizontal axis) and from miniaturized NexteraXT libraries (vertical axis). Point of equality is indicated by a dotted line, and values are presented for assemblies at a depth of 96 samples per lane (left panel) and at 384 samples per lane (right panel).

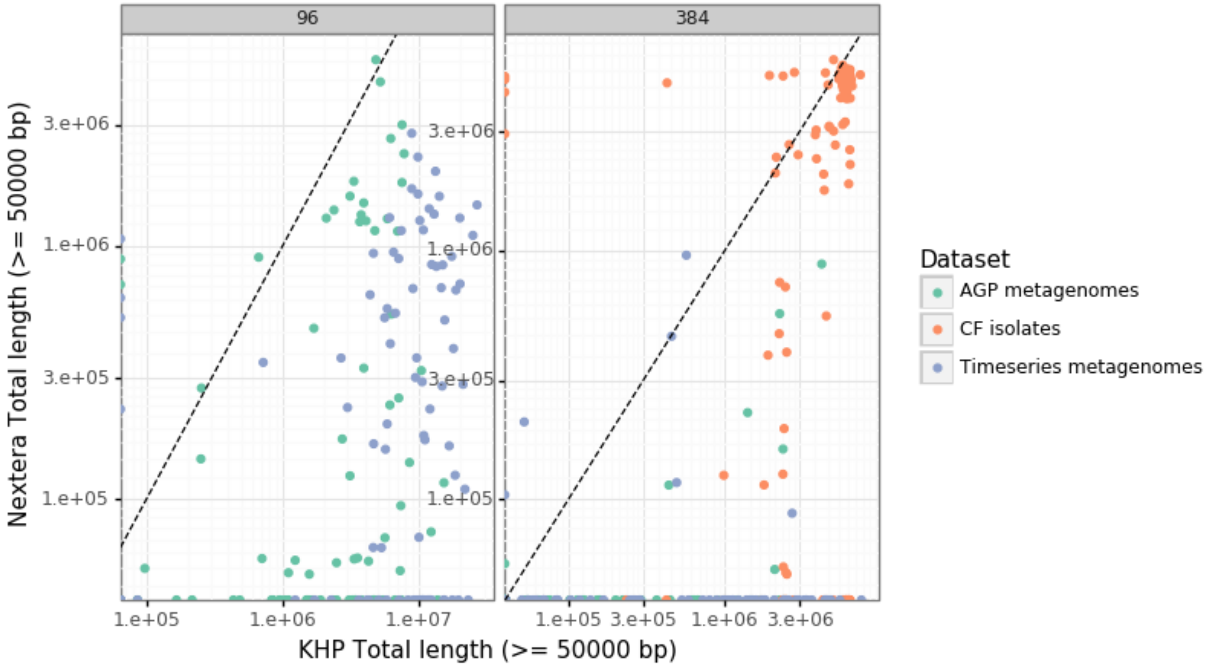

Figure S11. Total length of assembly in contigs ≥ 50 kbp for miniaturized libraries prepared from three different sample sets. Values for samples (points) assembled from miniaturized HyperPlus libraries (horizontal axis) and from miniaturized NexteraXT libraries (vertical axis). Point of equality is indicated by a dotted line, and values are presented for assemblies at a depth of 96 samples per lane (left panel) and at 384 samples per lane (right panel).

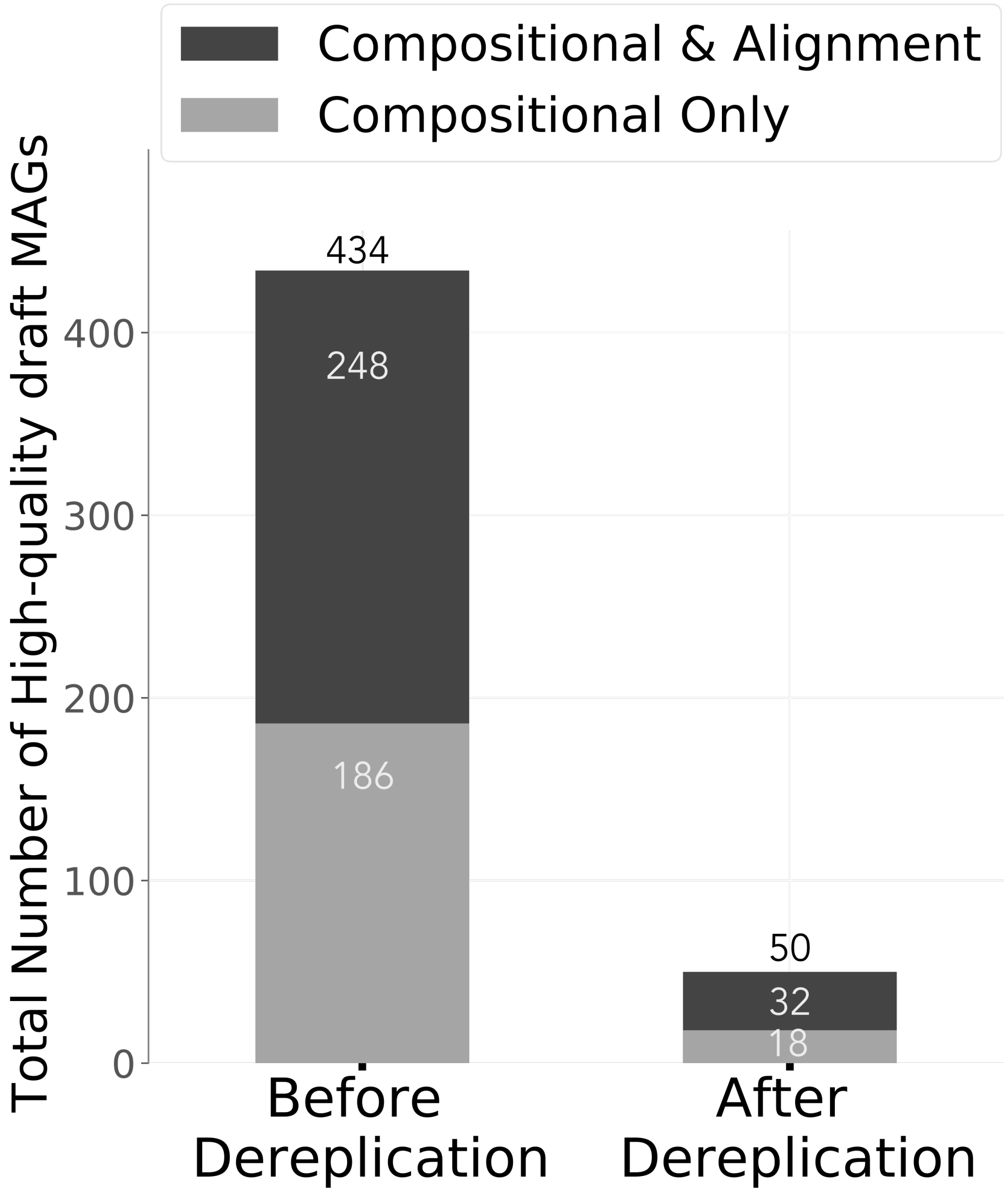

Figure S12. Results from bin dereplication with dRep.
