# Supplementary material for: Optimizing sequencing protocols for leaderboard metagenomics by combining long and short reads
Source: Genome Biol. 2019 Oct 31;20:226. doi: 10.1186/s13059-019-1834-9 (PMC6822431; doi:10.1186/s13059-019-1834-9)
Supplement: Supplementary file 2 — Additional file 2. Detailed miniaturized library prep protocol. Detailed, step-by-step protocol for miniaturized library preparation. [file 13059_2019_1834_MOESM2_ESM.docx]

Last Updated: 2019-03-26 (yyyy-mm-dd)

Robot: Mosquito large volume 500nl-40ul working volume
Library Prep: Kapa Hyper Plus with iTru adaptor strategy
Volume reduction: 1:10
Versions: 2 96-well Plate or 4 96-well Plates

Software: [library prep Jupyter notebook and Python package](https://github.com/tanaes/metagenomics_pooling_notebook)

#### Make echo compatible 384 source plate

*Transfer sample into an Echo compatible plate for acoustic droplet ejection dispensing.*

1. Reformat 4x 96-well plates into a 384PP echo plate.
   1. EpMotion
      1. 4 tip boxes 300uL f
      2. 60 uL transfer volume

#### Make sample plate map.

*Make a tab delimited text file that outlines sample locations in compressed plate.*

1. Use [this](https://docs.google.com/spreadsheets/d/1xPjB6iR3brGeG4bm2un4ISSsTDxFw5yME09bKqz0XNk/edit?usp=sharing) template to generate a .tsv file with sample locations
   1. Copy each 96 well plate map into corresponding compression slot in the “Compression” sheet
   2. Click on the “Map” sheet to reveal sample locations as a list.
   3. Finally, clic File > Download as > tab-separated values (.tsv)

#### Quantify & QC gDNA

*Quantify the concentration of gDNA per sample in order to normalize input.*

1. MiniPico De Verde Detection window 0.20 - 50 ng/µL
   1. Thaw Sample Plates (takes a while)
   2. Take out High Resolution DNA Standard Plate from fridge
      1. If Standard Plate is more than 1 month old make a new one.
         1. Dispense 100µL of λ DNA standard (invitrogen) in column 1 of a 384 PP echo plate. Write an appropriate plate label.
         2. Execute epMotion protocol following specified deck layout.
   3. Assay Recipe (per 384-well plate)
      1. 18,900 µL of Nuclease Free Water
      2. 1,000 µL 20x TE buffer
      3. 100 µL of PicoGreen Dye
   4. Calculate how many 384 sample plates you are quantifying and add a half plate for standards. (If quantifying 3x 384 plates you’ll need to Master Mix the MiniPico de Verde recipe times 3.5)
      1. 1 Plate + STD
         1. Add 28,350 µL of water to 30mL epMotion Reservoir
         2. Add 1,500 µL of 20x TE to Reservoir
         3. Add 150µL of PicoGreen Dye
         4. Mix well
      2. If running more that 1 sample plate you’ll need to master mix the Assay in a 100mL Reservoir and then redistribute into smaller reservoirs.
         1. For any additional plate you’ll dispense 19,100µL of MiniPico de Verde MM into a 30mL epMotion Reservoir.
   5. epMotion Automated Master Mix dispense:
      1. **One plate:**
         1. 50µL f Tips
         2. Reservoir Rack
            1. 19,100µL of MiniPico de Verde Assay in Reservoir
         3. 1 Black 384 Plates (Corning 3573)
            1. Label appropriately
      2. **One plate + Standard Curve:**
         1. 50µL f Tips
         2. Reservoir Rack
            1. 29,000µL of MiniPico de Verde Assay
         3. 2 Black 384 Plates (Corning 3573)
            1. Label appropriately
      3. **Two plates:**
         1. 50µL f Tips
         2. Reservoir Rack
            1. 19,100µL of MiniPico de Verde Assay in Reservoir Slot 1
            2. 19,100µL of MiniPico de Verde Assay in Reservoir Slot 2
         3. 2 Black 384 Plates (Corning 3573)
            1. Label appropriately
   6. Seal plates and spin down. Minimize light exposure.
   7. On Mosquitos transfer 1 µL of STD and Sample into their respective 384 Black Plates.
      1. **MiniPicoGreen_1xSample_Plate+STD_Plate**
      2. **MiniPicoGreen_2xSample_Plate**
      3. **MiniPicoGreen_STD_Plate**
   8. Seal plates. Vortex. Spin down.
   9. Read on BioTek Synergy HT reader
      1. Open Gen5 software
         1. File->New Task->Experiments->Create using an existing protocol->MiniPicoGreen_384
      2. Read new plate (STD plate is read first)
         1. After STD plate is read go to plate Statistics and write down the Mean of the Blank replicates. This value will be used to do blank subtractions on the rest of the plates that are being read.
         2.
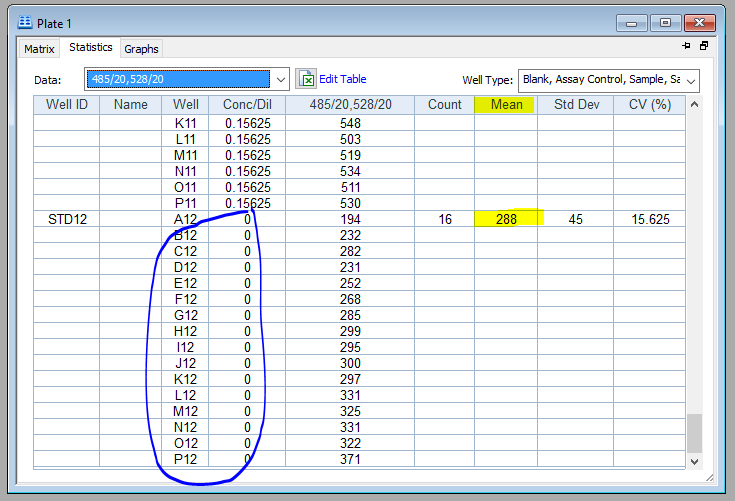

      3. Read next plate
         1. When you try to read the next plate you will be prompted to input the mean of Blank Wells with the following dialog:
            1.
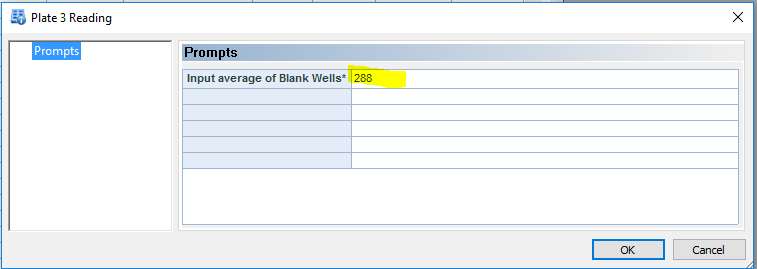

            2. Input the value you recorded.
         2. After plate is done reading you will be asked whether you want to export a Matrix with your results. If you choose yes you’ll get an Excel worksheet with the calculated concentrations per well in a matrix format.
         3. You can also choose to export a Table with the concentrations as a list
2. Make a pool of the gDNA plate and run a Genomic Tapestation

#### Echo Normalization of input DNA

*Make dilutions using echo instrument to ensure equal DNA input into Kapa Hyper Plus reaction (5 ng at 3.5 ul)*. *If gDNA concentration is not sufficient to reach 5 ng then input the maximum of 3.5 ul*

1. Generate an Input DNA Normalization Picklist.
   1. Use Qubit or Pico-green assay sample concentrations to normalize input DNA using Jupyter Notebook
2. Fill a 384PP echo compatible plate with 50 uL of molecular grade water per well.
3. Centrifuge both water and Sample Plate.
4. Write appropriate label for Eppendorf 384 PCR destination plate. (Colorless)
5. Open the DNA Input Normalization template that best fits your source plate
   1. Import the picklist prior to running the protocol. This is done by clicking Import Region Definitions… and selecting the generated picklist. The transfer map will appear in the Plate Reformat software. Delete the dummy transfer A1:A1 as this is no longer needed (delete both the source and destination plates associated with this)
      1. This method will prompt the user to save the protocol before execution. Save with a unique name that includes date and project information. *This method of protocol execution generates a descriptive Echo Transfer Report and a nice soft trail.*
6. Follow on screen directions for echo transfer.
   1. The proper sequence of source plates starts with the water plate and is followed by sample plates

#### Kapa HyperPlus Library Construction

##### Reagent Plate Master mix calculations

*Reagent volumes were optimized to process 768 samples per Kapa Hyper Plus kit taking into account dead volume at different liquid handling steps (both manual pipetting and automated liquid handling).*

| **iTru barcoding strategy** | | | | | |
| --- | --- | --- | --- | --- | --- |
|  | **# Samples** | | | | **Notes** |
|  | 1 (1x) | 1(1:10) | 192 | 384 |  |
| **Fragmentation mix** | 15 µL/sample | 1.5 µL/sample | 29 µL/ 1 column | 59 µL/ 1 column | **Make Fresh** |
| Frag enzyme | 10 µL | 1 µL | 315 µL | 630 µL | Keep Reagent plate on ice |
| Frag buffer | 5 µL | 0.5 µL | 158 µL | 315 µL | Thaw RT. Vortex |
| **End Repair & A-Tail** | 10 µL/sample | 1 µL/sample | 20 µL/ 1 column | 40 µL/ 1 column | **MM stable at RT for 24 hours** |
| ER&AT enzyme | 3 µL | 0.3 µL | 98 µL | 195 µL | Bump vortex or flick |
| ER&AT buffer | 7 µL | 0.7 µL | 228 µL | 455 µL | Thaw RT. Vortex |
| iTru Universal Stub (1.5 µM) |  | 360 nL transfer | 160 µL | 320 µL | Minimize freeze thaws |
| **Ligation Mix** | 45 µL/sample | 4.5 µL/samples | 35 µL/ 2 column | 46 µL/ 3 columns | **MM stable at RT for 24 hours** |
| Ligase Enzyme | 10 µL | 1 µL | 250 µL | 500 µL |  |
| Ligation buffer | 30 µL | 3 µL | 750 µL | 1500 µL | Thaw RT. Vortex |
| mol grade H20 | 5 µL | 0.5 µL | 125 µL | 250 µL |  |
| Elution Buffer | (PowerMag EB)  (10 mM Tris) |  |  |  |  |
| 80% EtOH |  |  | 500 mL | 500 mL | Make fresh |
| PCR Mastermix | 30 µL/sample | 5.5 µL/sample  (~ 1:5 ) | 32 µL/ 2 columns | 45 µL/ 3 columns |  |
| Mastermix |  | 5 µL |  |  |  |
| iTrue Primers [10µM] |  | 0.5 µL | Echo | Echo |  |

*The tables in the following page describe the reagent volumes per well that need to be aliquoted into the Reagent Plate. Colors match those presented in the Plate Master Mix calculations table.*

| **192 Samples (2 plates)** | | | | | | | | | | | | | | | | | | | | | | | | |
| --- | --- | --- | --- | --- | --- | --- | --- | --- | --- | --- | --- | --- | --- | --- | --- | --- | --- | --- | --- | --- | --- | --- | --- | --- |
|  | Frag  mix |  | ER &AT |  | Ligation mix | |  |  | PCR Mix | |  |  |  | | | |  |  |  |  |  | |  |  |
|  | 1 | 2 | 3 | 4 | 5 | 6 | 7 | 8 | 9 | 10 | 11 | 12 | 13 | 14 | 15 | 16 | 17 | 18 | 19 | 20 | 21 | 22 | 23 | 24 |
| A | 29 |  | 20 |  | 35 | 35 |  |  | 32 | 32 |  |  |  |  |  |  |  |  |  |  |  |  |  |  |
| B | 29 |  | 20 |  | 35 | 35 |  |  | 32 | 32 |  |  |  |  |  |  |  |  |  |  |  |  |  |  |
| C | 29 |  | 20 |  | 35 | 35 |  |  | 32 | 32 |  |  |  |  |  |  |  |  |  |  |  |  |  |  |
| D | 29 |  | 20 |  | 35 | 35 |  |  | 32 | 32 |  |  |  |  |  |  |  |  |  |  |  |  |  |  |
| E | 29 |  | 20 |  | 35 | 35 |  |  | 32 | 32 |  |  |  |  |  |  |  |  |  |  |  |  |  |  |
| F | 29 |  | 20 |  | 35 | 35 |  |  | 32 | 32 |  |  |  |  |  |  |  |  |  |  |  |  |  |  |
| G | 29 |  | 20 |  | 35 | 35 |  |  | 32 | 32 |  |  |  |  |  |  |  |  |  |  |  |  |  |  |
| H | 29 |  | 20 |  | 35 | 35 |  |  | 32 | 32 |  |  |  |  |  |  |  |  |  |  |  |  |  |  |
| I | 29 |  | 20 |  | 35 | 35 |  |  | 32 | 32 |  |  |  |  |  |  |  |  |  |  |  |  |  |  |
| J | 29 |  | 20 |  | 35 | 35 |  |  | 32 | 32 |  |  |  |  |  |  |  |  |  |  |  |  |  |  |
| K | 29 |  | 20 |  | 35 | 35 |  |  | 32 | 32 |  |  |  |  |  |  |  |  |  |  |  |  |  |  |
| L | 29 |  | 20 |  | 35 | 35 |  |  | 32 | 32 |  |  |  |  |  |  |  |  |  |  |  |  |  |  |
| M | 29 |  | 20 |  | 35 | 35 |  |  | 32 | 32 |  |  |  |  |  |  |  |  |  |  |  |  |  |  |
| N | 29 |  | 20 |  | 35 | 35 |  |  | 32 | 32 |  |  |  |  |  |  |  |  |  |  |  |  |  |  |
| O | 29 |  | 20 |  | 35 | 35 |  |  | 32 | 32 |  |  |  |  |  |  |  |  |  |  |  |  |  |  |
| P | 29 |  | 20 |  | 35 | 35 |  |  | 32 | 32 |  |  |  |  |  |  |  |  |  |  |  |  |  |  |
| **384 Samples (4 plates)** | | | | | | | | | | | | | | | | | | | | | | | | |
|  | 1 | 2 | 3 | 4 | 5 | 6 | 7 | 8 | 9 | 10 | 11 | 12 | 13 | 14 | 15 | 16 | 17 | 18 | 19 | 20 | 21 | 22 | 23 | 24 |
| A | 58 |  | 40 |  | 45 | 45 | 45 |  | 45 | 45 | 45 |  |  |  |  |  |  |  |  |  |  |  |  |  |
| B | 58 |  | 40 |  | 45 | 45 | 45 |  | 45 | 45 | 45 |  |  |  |  |  |  |  |  |  |  |  |  |  |
| C | 58 |  | 40 |  | 45 | 45 | 45 |  | 45 | 45 | 45 |  |  |  |  |  |  |  |  |  |  |  |  |  |
| D | 58 |  | 40 |  | 45 | 45 | 45 |  | 45 | 45 | 45 |  |  |  |  |  |  |  |  |  |  |  |  |  |
| E | 58 |  | 40 |  | 45 | 45 | 45 |  | 45 | 45 | 45 |  |  |  |  |  |  |  |  |  |  |  |  |  |
| F | 58 |  | 40 |  | 45 | 45 | 45 |  | 45 | 45 | 45 |  |  |  |  |  |  |  |  |  |  |  |  |  |
| G | 58 |  | 40 |  | 45 | 45 | 45 |  | 45 | 45 | 45 |  |  |  |  |  |  |  |  |  |  |  |  |  |
| H | 58 |  | 40 |  | 45 | 45 | 45 |  | 45 | 45 | 45 |  |  |  |  |  |  |  |  |  |  |  |  |  |
| I | 58 |  | 40 |  | 45 | 45 | 45 |  | 45 | 45 | 45 |  |  |  |  |  |  |  |  |  |  |  |  |  |
| J | 58 |  | 40 |  | 45 | 45 | 45 |  | 45 | 45 | 45 |  |  |  |  |  |  |  |  |  |  |  |  |  |
| K | 58 |  | 40 |  | 45 | 45 | 45 |  | 45 | 45 | 45 | i |  |  |  |  |  |  |  |  |  |  |  |  |
| L | 58 |  | 40 |  | 45 | 45 | 45 |  | 45 | 45 | 45 |  |  |  |  |  |  |  |  |  |  |  |  |  |
| M | 58 |  | 40 |  | 45 | 45 | 45 |  | 45 | 45 | 45 |  |  |  |  |  |  |  |  |  |  |  |  |  |
| N | 58 |  | 40 |  | 45 | 45 | 45 |  | 45 | 45 | 45 |  |  |  |  |  |  |  |  |  |  |  |  |  |
| O | 58 |  | 40 |  | 45 | 45 | 45 |  | 45 | 45 | 45 |  |  |  |  |  |  |  |  |  |  |  |  |  |
| P | 58 |  | 40 |  | 45 | 45 | 45 |  | 45 | 45 | 45 |  |  |  |  |  |  |  |  |  |  |  |  |  |

#####
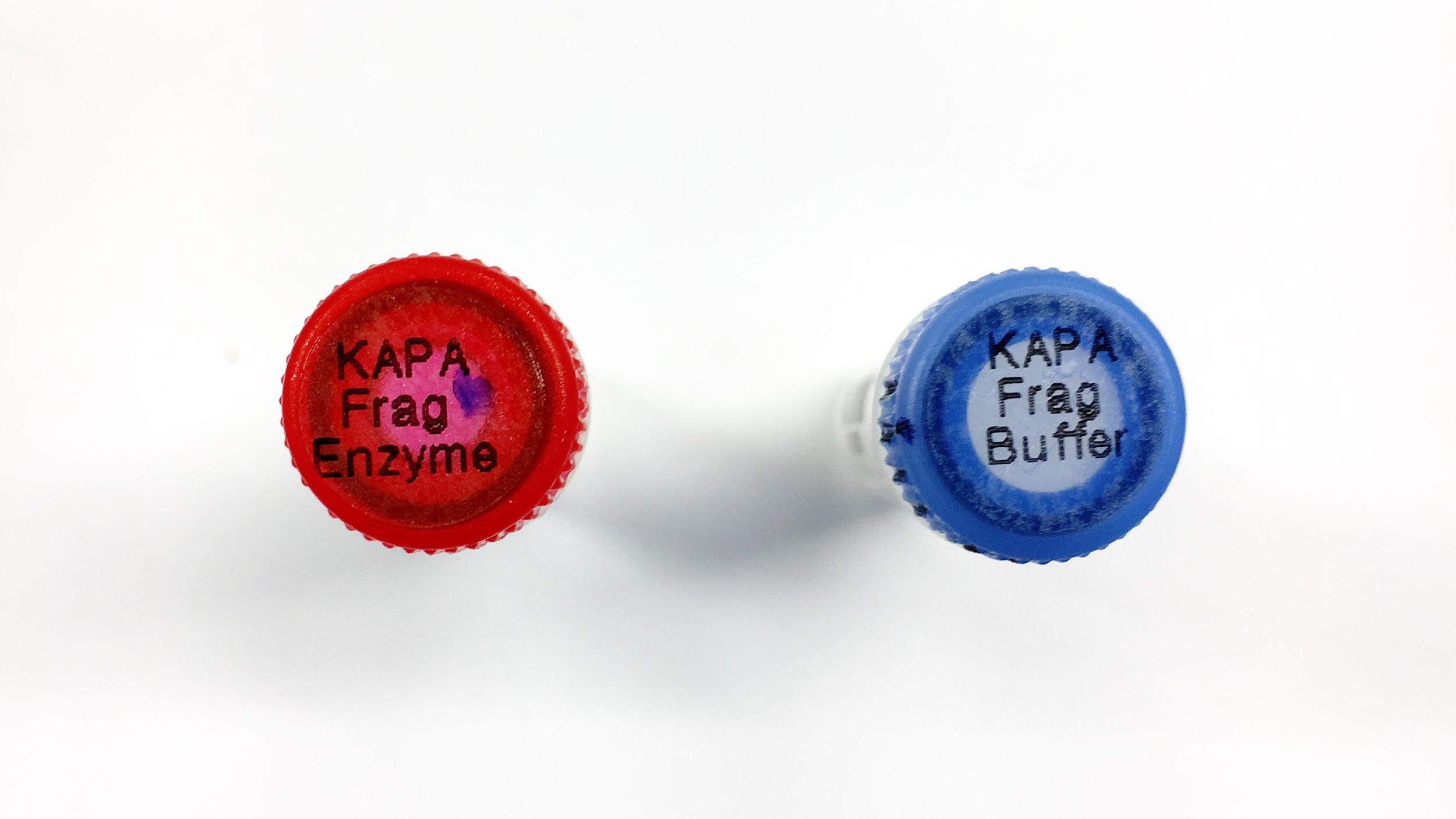
1. Fragment reaction


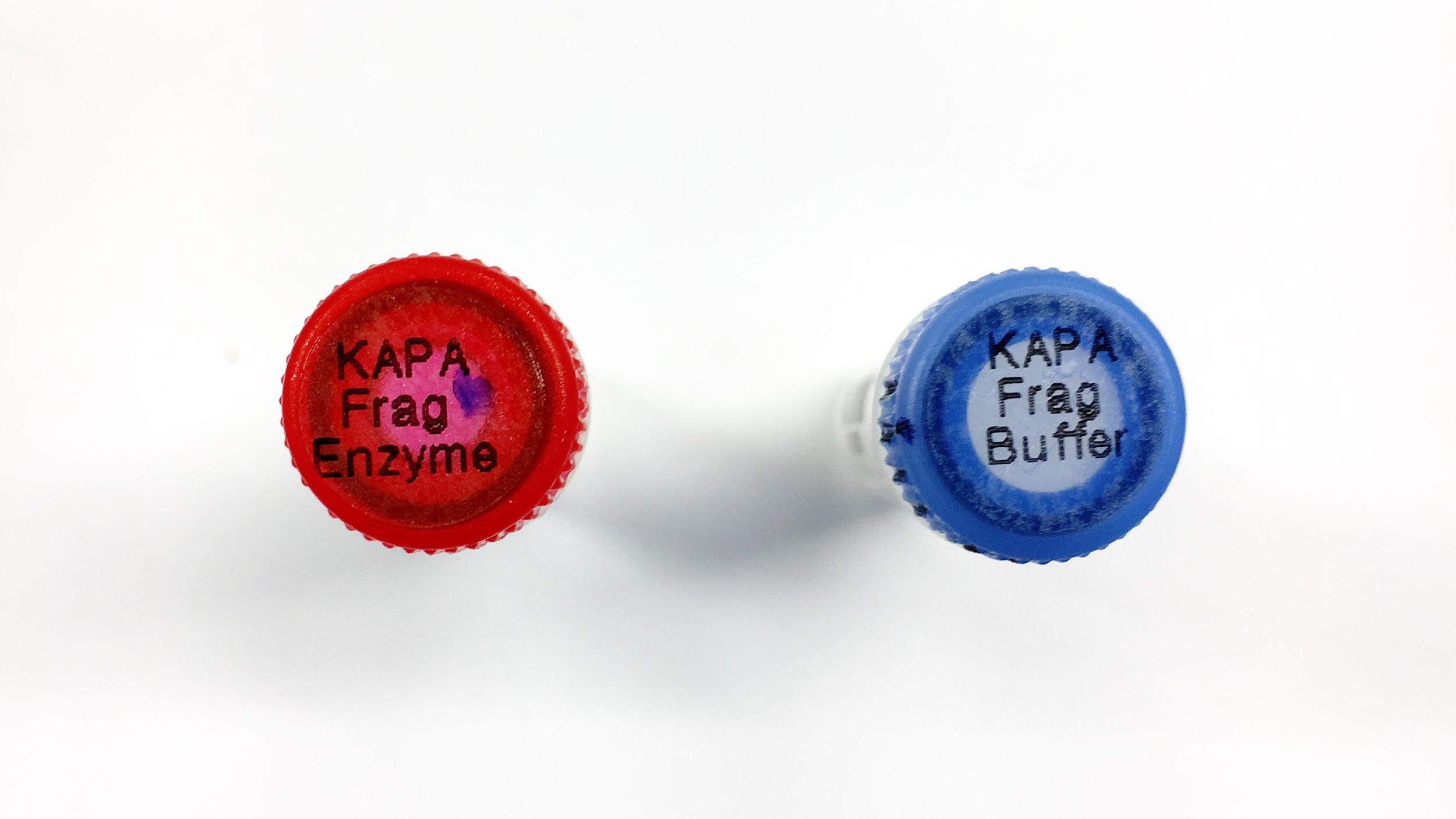
(KEEP REAGENTS ON ICE)

A. Thaw fragment buffer, vortex 10s full speed
B. Make premix using eppendorf P1000

192 samples: 315 ul fragment enzyme + 158 ul fragment buffer

384 samples: 630 ul fragment enzyme + 315 ul fragment buffer

C. Add premix (by hand) to 384 TTP_LVSD plate ‘Reagent Plate’ [held on 4C cold block/ice]

192 samples: add 29 ul to 16 wells in column 1 (using P100)

384 samples: add 58 ul to 16 wells in column 1 (using P100)


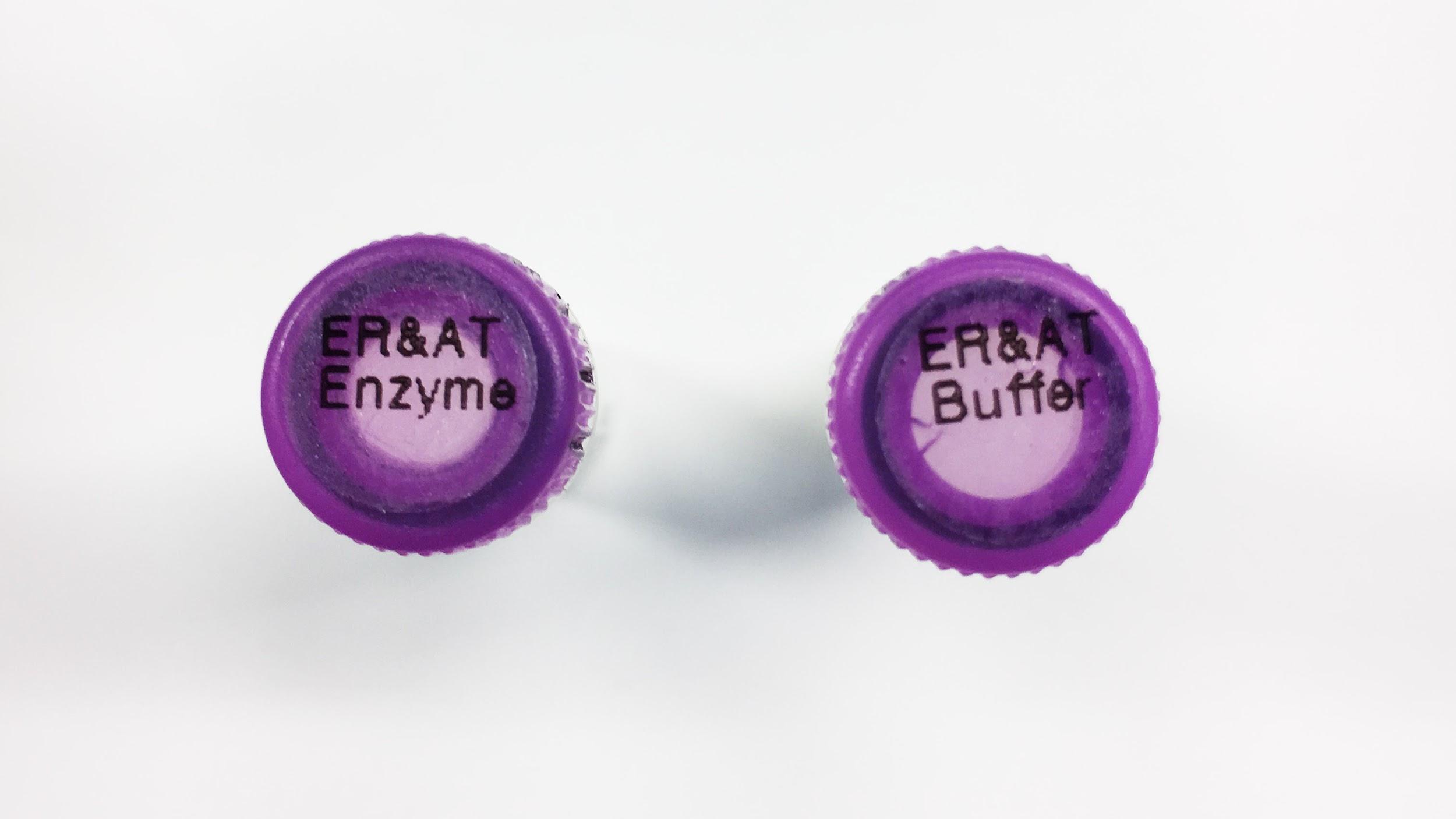
D. Start mosquito protocol
*#transfers 1.5ul of fragment enzyme-buffer mix to ‘Sample Plate’ 384 well eppendorf.*
E. Seal plate, place on precooled thermocycler at 37 ^o^C for 20 min, followed by 4 ^o^C hold

##### 2. End-repair & A-tail

A. Thaw A-tail/End-repair buffer at RT 15 min, vortex 10s full speed
B. Make premix using eppendorf P1000

192 samples: 98 ul ER&AT enzyme + 228 ul ER&AT buffer

384 samples: 195 ul ER&AT enzyme + 455 ul ER&AT buffer

C. Add premix (by hand) to 384 TTP_LVSD plate ‘Reagent Plate’

192 samples: 20 ul to 16 wells in column 3 (using P20)

384 samples: 40 ul to 16 wells in column 3 (using P100)

D. Start mosquito protocol
*#transfers 1ul of end repair-a-tail enzyme-buffer mix to ‘Sample Plate’ 384 well eppendorf*
E. Seal plate, place on thermocycler at 65 ^o^C for 30 min, 4 ^o^C hold

##### 3. Adaptor ligation

A. Thaw adaptor ligase buffer RT 15 min (vortex really well), ligase enzyme 4 ^o^C, sigma water UV treated, and iTru Universal Stub adaptor
B. Add iTru Universal Stub adaptor to Echo LDV Plate

384 samples: 11.5 µL to 16 wells in column 1 (using P20)


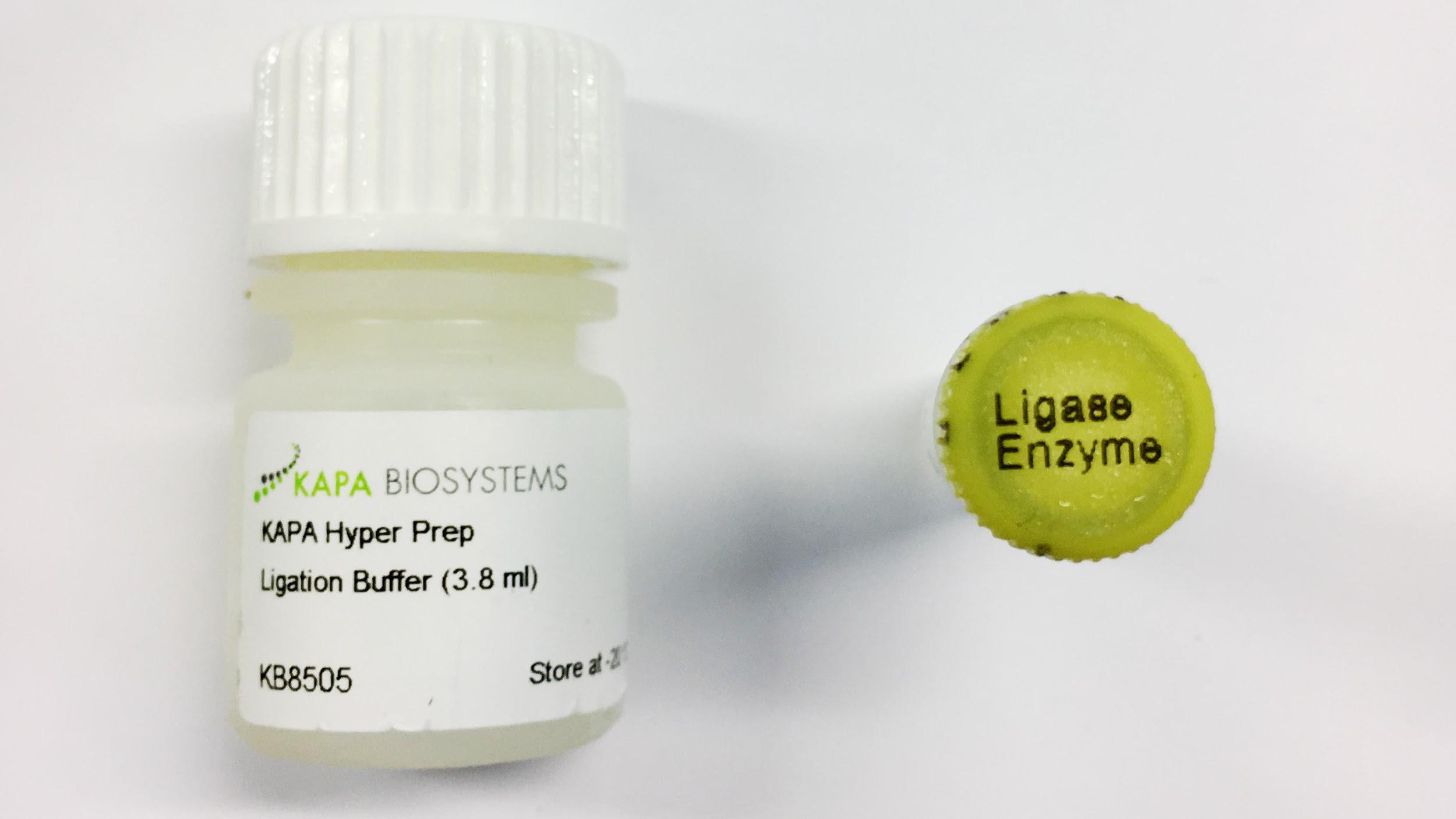
C. Make Adaptor ligation premix using eppendorf P1000

192 samples: 250 ul DNA ligase enz + 750 ul ligase buffer + 125 ul H20

384 samples: 500 ul DNA ligase enz + 1500 ul ligase buffer + 250 ul H20

D. Add premix (by hand) to 384 TTP_LVSD plate ‘Reagent Plate’

192 samples: 35 ul to 16 wells in column 5 and 6 (using P100)

384 samples: 45 ul to 16 wells in columns 5, 6, and 7 (using P100)

Start mosquito protocol
*#transfers 4.5 ul of adaptor lig mix to ‘Sample Plate’ 384 well eppendorf*
E. incubate RT or 20 ^o^C for 1hr

Possible stopping point (you can let plate incubate overnight at 4^o^C after RT incubation of up to 2 hrs)

#####
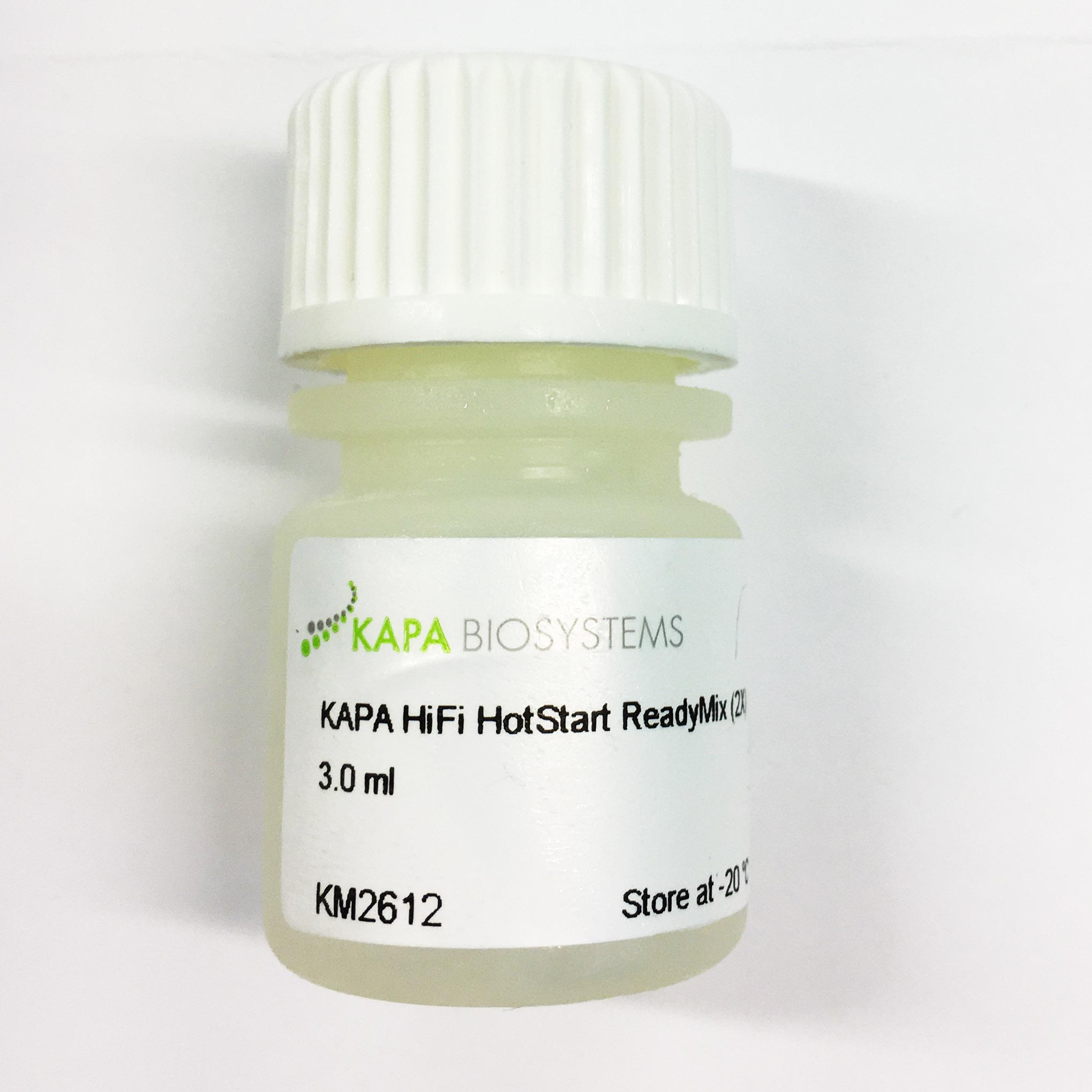
 4. PCR Setup

RUN WHILE ADAPTOR LIGATION INCUBATES

1. Thaw PCR HiFi HotStart Readymix and iTru Primer plate
2. Add PCR Mastermix (by hand) to 384 TTP_LVSD plate ‘Reagent Plate’

192 samples: 32 ul to 16 wells in columns 9-10

384 samples: 45 ul to 16 wells in columns 9-11

1. Appropriately label 384 Eppendorf destination plate (Green). (PCR Ready Plate)
2. Start mosquito protocol

*#transfers 5.0 µL of PCR MM into final 384 Destination plate (PCR Ready Plate)*

1. Transfer unique dual indexing primers to each well using the Echo.
   1. Generate index picklist with Jupyter Notebook
      1. Take a number from ticket dispenser
      2. Input number into “Plate Counter” field in notebook and retain for records.
      3. Use generated index picklist to transfer the unique combination of primers to PCR plate.
   2. Transfer primers with the Echo
      1. Import the picklist prior to running the protocol. This is done by clicking Import Region Definitions… and selecting the generated picklist. The transfer map will appear in the Plate Reformat software. Delete the dummy transfer A1:A1 as this is no longer needed (delete both the source and destination plates associated with this)
         1. This method will prompt the user to save the protocol before execution. Save with a unique name that includes date and project information. *This method of protocol execution generates a descriptive Echo Transfer Report and a nice soft trail.*
      2. i5 primers will be dispensed first
      3. i7 primers will be dispensed second

*#transfers 0.25 µL of forward primer and 0.25µL of reverse primer in unique combinations.*

##### 5. Adaptor cleanup

1. Resuspend AMPure XP beads (120s vortex max speed)
2. Dispense appropriate amount of beads into 30mL EpMotion reservoir. (Click “Use required minimum volumes” on Application Runner Wizard)

192 Samples: 1985 µL of AMPure Beads (not yet tested 07-12-2017)

384 Samples: 3425 µL of AMPure Beads

1. On EpMotion, add 7.5 ul AMPure XP beads to 384 Sample Plate
2. Seal plate
3. Vortex plate for 2 min
4. Spin down at 1000 RCF for 1 min to let beads settle and normalize the meniscus
5. Prepare 500mL Bottle of 80% EtOH (freshly made)
6. Run Water dispense in BlueCat Washer
   1. 384 Eppendorf Plate
   2. Water dispense protocol overview
      1. Prime White Channel (Internal Volume)
      2. Dispense 40 µL at pressure level 3.
7. Seal plate
8. Spin down at 1000 RCF for 1 min
9. Run Bead cleanup protocol in BlueCat Washer
   1. 384 Sample Plate
   2. Bead cleanup protocol overview
      1. Prime Green Channel (Internal Volume)
      2. Evacuate supernatant
      3. Wash beads once with 30 µL of 80% Ethanol (Green) at pressure level 3
10. Immediately run Elution Recovery protocol on Mosquito.
    1. Elution recovery overview
       1. Aliquot 7 µL of water from 384 Water Plate into 384 Sample Plate with Beads
       2. Seal plate. Resuspend beads by vortexing and waving magnet around bottom of wells. (**user intervention**)
       3. Spin down at 1000 RCF for 1 min to let beads settle and normalize the meniscus.
       4. Aliquot 4.5 µL of cleaned adapted library from 384 Eppendorf Plate into PCR Ready plate.

**NOTE: ELUTION (4.5µL ) WILL GO INTO THE PCR READY PLATE MADE IN STEP IV.**

Spin down plate and PCR for 15 cycles.

| 384-well | 15 PCR cycles | | |  |  |
| --- | --- | --- | --- | --- | --- |
| 98 ^o^C | 98 ^o^C | 60 ^o^C | 72 ^o^C | 72 ^o^C | 4 ^o^C |
| 45 s | 15 s | 30 s | 30 s | 1 min | hold |

#### Normalized Pooling

##### 1. Post amplification bead cleanup.

1. Resuspend AMPure XP beads (120s vortex max speed)
2. Dispense appropriate amount of beads into 30mL EpMotion reservoir. (Click “Use required minimum volumes” on Application Runner Wizard)

192 Samples: 1985 µL of AMPure Beads (not yet tested 07-12-2017)

384 Samples: 3425 µL of AMPure Beads

1. On EpMotion, add 7.5 ul AMPure XP beads to Sample Plate
2. Seal plate
3. Vortex plate for 2 min
4. Spin down at 1000 RCF for 1 min to let beads settle and normalize the meniscus
5. Prepare 500mL Bottle of 80% EtOH (freshly made)
6. Run Water dispense in BlueCat Washer
   1. 384 Eppendorf Plate
   2. Water dispense protocol overview
      1. Prime White Channel (Internal Volume)
      2. Dispense 40 µL at pressure level 3.
7. Seal plate
8. Spin down at 1000 RCF for 1 min
9. Run Bead cleanup protocol in BlueCat Washer
   1. 384 Library Plate
   2. Bead cleanup protocol overview
      1. Prime Green Channel (Internal Volume)
      2. Evacuate supernatant
      3. Wash beads once with 30 µL of 80% Ethanol (Green) at pressure level 3
10. Run Clean Library recovery protocol on Mosquito.
    1. Clean Library recovery overview
       1. Aliquot 10 µL of water from 384 Eppendorf Plate into 384 Library Plate
       2. Seal plate. Resuspend beads by vortexing and waving magnet around bottom of wells. (**user intervention**)
       3. Spin down at 1000 RCF for 1 min to let beads settle and normalize the meniscus.
       4. Aliquot 9 µL of cleaned adapted library from 384 Library Plate into Echo LDV plate. (Library Plate for storage)

##### 2. Library Quantification

*Quantify Library concentration per sample in order to normalize sequencing coverage.*

1. MiniPico De Verde Detection window 0.20 - 50 ng/µL
   1. Thaw Sample Plates (takes a while)
   2. Take out High Resolution DNA Standard Plate from fridge
      1. If Standard Plate is more than 1 month old make a new one.
         1. Dispense 100µL of λ DNA standard (invitrogen) in column 1 of a 384 PP echo plate. Write an appropriate plate label.
         2. Execute epMotion protocol following specified deck layout.
      2. Assay Recipe (per 384-well plate)
      3. 18,900 µL of Nuclease Free Water
      4. 1,000 µL 20x TE buffer
      5. 100 µL of PicoGreen Dye
   3. Calculate how many 384 sample plates you are quantifying and add a half plate for standards. (If quantifying 3x 384 plates you’ll need to Master Mix the MiniPico de Verde recipe times 3.5)
      1. 1 Plate + STD
         1. Add 28,350 µL of water to 30mL epMotion Reservoir
         2. Add 1,500 µL of 20x TE to Reservoir
         3. Add 150µL of PicoGreen Dye
         4. Mix well
      2. If running more that 1 sample plate you’ll need to master mix the Assay in a 100mL Reservoir and then redistribute into smaller reservoirs.
         1. For any additional plate you’ll dispense 19,100µL of MiniPico de Verde MM into a 30mL epMotion Reservoir.
   4. epMotion Automated Master Mix dispense:
      1. **One plate:**
         1. 50µL f Tips
         2. Reservoir Rack
            1. 19,100µL of MiniPico de Verde Assay in Reservoir
         3. 1 Black 384 Plates (Corning 3573)
            1. Label appropriately
      2. **One plate + standard curve:**
         1. 50µL f Tips
         2. Reservoir Rack
            1. 29,000µL of MiniPico de Verde Assay
         3. 2 Black 384 Plates (Corning 3573)
            1. Label appropriately
      3. **Two plates:**
         1. 50µL f Tips
         2. Reservoir Rack
            1. 19,100µL of MiniPico de Verde Assay in Reservoir Slot 1
            2. 19,100µL of MiniPico de Verde Assay in Reservoir Slot 2
         3. 2 Black 384 Plates (Corning 3573)
            1. Label appropriately
   5. Seal plates and spin down. Minimize light exposure.
   6. On Mosquitos transfer 1 µL of STD and Sample into their respective 384 Black Plates.
      1. **MiniPicoGreen_1xSample_Plate+STD_Plate**
      2. **MiniPicoGreen_2xSample_Plate**
      3. **MiniPicoGreen_STD_Plate**
   7. Seal plates. Vortex. Spin down.
   8. Read on BioTek Synergy HT reader
      1. Open Gen5 software
         1. File->New Task->Experiments->Create using an existing protocol->MiniPicoGreen_384
      2. Read new plate (STD plate is read first)
         1. After STD plate is read go to plate Statistics and write down the Mean of the Blank replicates. This value will be used to do blank subtractions on the rest of the plates that are being read.
         2.
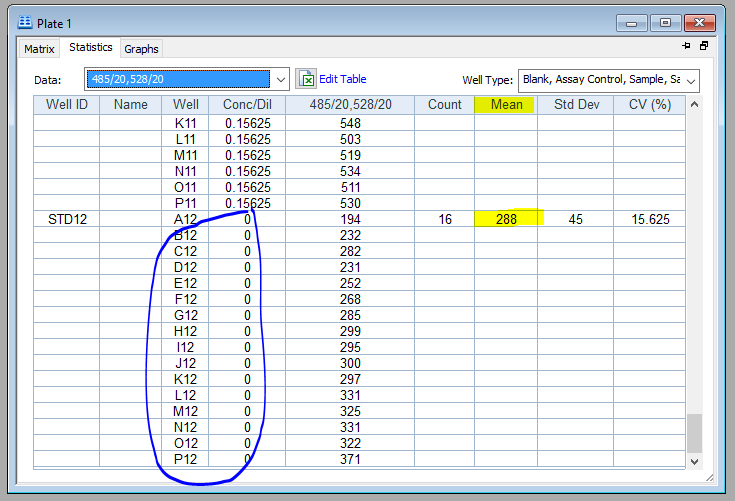

      3. Read next plate
         1. When you try to read the next plate you will be prompted to input the mean of Blank Wells with the following dialog:
            1.
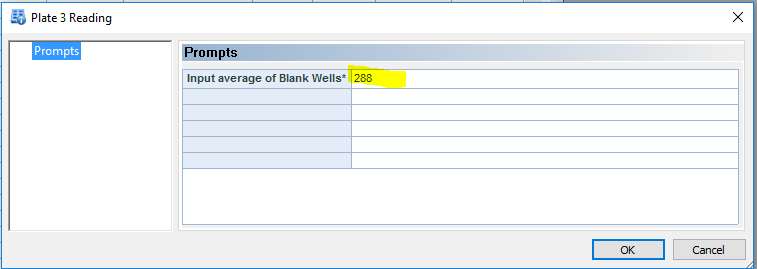

            2. Input the value you recorded.
         2. After plate is done reading, choose to export a Table with the concentrations as a list
            1. Exporting data as a list will output a file that you can feed the jupyter notebook to obtain a normalized pooling picklist for the echo.

##### 3. Jupyter notebook normalized picklist generation.

1. Read in MiniPico Library concentrations with Jupyter Notebook
   1. Step 4.1-4.2 in Notebook
2. Calculate best pooling values for normalization. We stereotypically normalize using the minvol approach.
   1. To estimate best pooling values for normalization you can follow these steps.
      1. Visualize sample concentration across whole plate.
         1. Steps 4.4 in Notebook
      2. Estimate average concentration of blank samples and use this value for the **floor_conc** parameter in the minvol normalization module (4.3)
      3. Run minvol normalization module with default **floor_vol**  and **total_nmol** parameters.
      4. Estimate pool concentration and volume and visualize pooling volumes across whole plate.
         1. We typically pool 50-100 µL. Adjust **total_nmol** parameter to increase or decrease the size of the whole pool or adjust **floor_vol** to pool more/less volume from blank/failed samples.
         2. Check the histogram of pooling volumes. Make sure blank/failed samples are being pooled at a volume comparable to the 10th percentile of real samples (that is, the volume pooled for highly concentrated samples).
   2. Generate picklist with notebook.
   3. Pool on echo into a 384 Eppendorf Plate.

##### 4. QC and process pool for sequencing.

##### Run Genomic Tapestation on pool. (We are trying to optimize our pooling procedure and we need concentration data on pooling estimates accuracy)

##### Concentrate pool down to 25µL using QIAGEN PCR Cleanup column.

##### Run Genomic Tapestation on pool.

##### Pippin Size select 300-700 bp.

##### Run High Sensitivity Tapestation size selected pool.
