# Supplementary material for: Optimizing sequencing protocols for leaderboard metagenomics by combining long and short reads
Source: Genome Biol. 2019 Oct 31;20:226. doi: 10.1186/s13059-019-1834-9 (PMC6822431; doi:10.1186/s13059-019-1834-9)
Supplement: Supplementary file 3 — Additional file 3. Review history. [file 13059_2019_1834_MOESM3_ESM.docx]

Review History

**First round of review**

**Reviewer 1**

**Were you able to assess all statistics in the manuscript, including the appropriateness of statistical tests used?** Yes, see comments below.

**Were you able to directly test the methods?** No.

**Comments to author:**

Sandres et al., basically describe a new protocol to allow inexpensive and low DNA input library prep for Illumina shotgun metagenome sequencing of microbiomes. This represents the most novel part of the paper, with the caveat that similar low input protocols have been described and used by others and were not mentioned here (see below). The remaining results were already known or highlight anticipated such as the limitations (but also the advantages in terms of low DNA input) of the Nextera protocol in terms of assembly quality. As such, the paper appears to be a biotech technique with no new biological or evolutionary insights into the microbiome of humans or other hosts/habitats. It appears that the authors have an interesting application of the technique to mice microbiomes but opted to not include the biological part of that work here. This reviewer thinks that overall the method is solid and well executed and possibly will be used by others in the field. However, the reviewer would also like to see some direct comparisons or at least discussion to other similar miniaturized protocols used by others such as the JGI or the MIT groups (e.g., Paul Blainey and his team). The text is not always well-written or well-connected, and several ambiguities exist throughout (see also specific comments below). Some editorial editing will be need. Finally, mock community is the only way to do this type of project/work robustly and highly accurately, and it is not as labor intensive as the authors imply in the abstract (see also below). There was nowhere in the manuscript any data/results to suggest that what the authors did here could not have been done with a mock sample. For instance, the example provided in the middle of page 9 is a clear case where only mock samples can do this well. Even the long read Illumina technology proposed as an alternative is error-prone and costly that not many labs can afford it (plus Illumina has discontinued them due probably to these limitations). I suggest revising the justification for the study to not sound so critical of mock samples and given a misleading view. The accession numbers for the sequencing data reported need to be made available before acceptance of the manuscript. 

Minor-specific comments. 

Ln 33-38, pg 5. I agree with the need, but the mock work is overly criticized in my view here. If one wants to do a real mock, one could just add DNA or cells of interest on a real reference stool sample. It is a bit more labor intensive but the alternative proposed here is not much cheaper either. I would not criticize mock work the way that the authors did and my suggestion to them is to lower the tone on this or completely remove these part.

Ln 18-22, pg 7. I believe this result (and others mentioned elsewhere) is consistent with what Illumina has published. If not, please make it clear. Also, I am not sure the comparison per cost makes scientific sense; I think a comparison based on same number of sequences (total bp) makes more sense (and say what is the associated cost per bp).

Ln 34, pg 7. I am a bit surprised that the authors used one of the least performing binning algorithm out there. MaxBin, MetaBAT and other more recent algorithms have been shown to do better job that CONCOCT in general. See also the CAMI challenge papers for details. It seems this was used in terms of convenience with the Anvi'o pipeline and likely did not affect results much though. Some discussion of this topic would have been useful, especially in terms of instability in CONCOCT or how fine tuning of its parameters could affect the assembly of datasets from different library creation methods differently. 

Pg 8. Why there is an almost empty sheet on page 8? Formatting issues?

Ln 4-9, pg 9. TruSeqNano was compared for the 5 best assembled genomes; why NexteraXT on 40? Seems inconsistent. Also, I suspect that some of the results may be due to higher similarity of these library making methods to the long-read method. Can the authors prove that this is not the case here? This seems to be critical information that is missing.

Ln25-50, pg 9. This analysis is much better done with a mock (known) genome for instance on page 9. 

Ln34-35. pg 10. This is interesting but I think it is better for a more specialized journal on biotech techniques. In any case, what is the cost for the required infrastructure e.g., for liquid handlers? This should be discussed for the estimate of 7$/sample to not be misleading.

ln 20, pg 12. "commonly yielded ≈ megabases". Is "≈" a typo or what?

Ln 53. BinS (typo)

Ln 24-25, pg 14. bins and MAGs are the same thing; yet, it is a bit confusing how MAGs were used here.

Ln 24, pg 15. rRNA GENE profiling. (gene is missing)

**Reviewer 2**

**Were you able to assess all statistics in the manuscript, including the appropriateness of statistical tests used?** Yes. No additional comments.

**Were you able to directly test the methods?** No.

**Comments to author:**

The paper is broadly concerned with developing production scale metagenomes for large sample sets. This is key important problem in contemporary microbiome research, and this paper makes some new contributions. The authors first consider how to define reference genomes using multiple-single sample assemblies constructed using synthetic long read data, obtaining reference genomes from most abundant community members: an approach they describe as 'leaderboard metagenomics' (although this strikes as a piece of unnecessary jargon).  The second part of the paper evaluates the use of high throughput library constructions in the relation to genome recoverability. The data and findings of the second half of the work are particularly valuable in moving toward to production scale genome-resolved metagenomics.

The concept developed here is roughly that a community is sampled that has variable abundance dynamics, so it is plausible that by using sufficiently large samples, one can take advantage of many observations to recover a particular member genome with accuracy, and so obtain a cohort of high quality genomes from the community. The approach is actually implicit in the dRep workflow from Banfield's group (Olm; PMID: 28742071), in which cognate bins from multiple binned metagenome assemblies are identified and the best instantiation retained as a 'dereplicated' reference, and this paper should be mentioned in the Discussion. The approach was also used in 2014 by Nielsen et al (which the authors cite) in a gene-centric partial genome recovery method described as 'canopies' (and which has not been developed much further to the best of my knowledge). As the authors already highlight, the issue is related to the use of co-assemblies, although the relative advantages and disadvantages of co-assemblies versus multiple single sample assemblies are still being worked out. 

Specific comments:
p5, line 13. "...can be used for mapping based analysis of less abundant species and strain variants within each sample". Yes, they can but there will be genomes which cannot be well recovered and that do not have conspecifics in the most abundant recovered set, which be missed by this approach. Ultimately it depends on how complex the community is in terms of number of species (richness), evenness (high evenness communities will be more difficult to analyse) and genetic/genomic diversity, and some mention should be made of these constraints in the Discussion; see also note below.

p5, line 53-54. "...apply to other highly accurate long read technologies as they emerge". Recent work on long read MAG analysis should be cited here (Arumugam, 2019; PMID:30992083 and Nicholls, 2019; PMID:31089679 and Somerville PMID:31238873). 

p9, paragraph 2, line 24. Does "mismatch" simply refer to the total number of observed alignment mismatches between the test assemblies the TSLR references?. I may have missed it, but as far as I can tell this is not explained anywhere.

p9 paragraph 2, line29. "....with lower coverage bins...capacity for internal error correction (Fig 2)." is very unclear. Please try and improve the clarity of this sentence. 

p12, last paragraph. What is unclear here is the degree of redundancy in the set of bins recovered these analyses. Would recommend the authors apply the dRep workflow to determine the number of secondary clusters, which represents the actual number of dereplicated genomes present. Additionally, the total number of bins detected in low, medium and high quality levels (i.e. MINIMAG criteria of Bowers) should be reported.

p13-p15. Finally, there are aspects to the Discussion that need some balance. One impression is that this paper may implicitly convey to the non-expert reader is that it is only top most abundant taxa that are important, and secondly that communities will be variable enough over time that by using repeated surveys that individual member genomes will be able to be recovered across the a reasonably broad abundance range. Neither assumption is demonstrated to be the case in this analysis, and my concern is that this will get misinterpreted and mis-cited by naive or over-enthusiastic practitioners. I agree that given sufficient samples of the same community type the concept of "expand[ing]...organically" (p14, line26) is logical and compelling, however, this is may be feasible only for communities of limited complexity, and it remains unclear whether repeated observations, coupled with MAG analysis as performed here, we be able to resolve more elusive member genomes of complex communities such as activated sludge or soil. In part this opinion also relates to my comment above about the unnecessary terminology of 'leaderboard metagenomics'.     

p13, line 32. As stated above, such long read analyses have already appeared in the literature, so they should be cited here (see note about p5, line 53-54)

p16. Methods, line 4. There needs to be a lot more detail provided here about these sample. What was the duration between sampling? Can the authors please list the details of the IRB approval as per standard practice. When were the additional samples referred to line 29 of the same page taken?

p16, line 52."we modified SPAdes". Can you provide some more detail? Is this covered by the material in the subsequent sentence starting "In particular..."? Is this code available?

p17, p41. "manually refined". Do the authors have any logs or outputs from anvi-refine to show the extent of these procedures?

**Authors Response**

**Point-by-point responses to the reviewers’ comments:**

Reviewer #1: ===

Sanders et al., basically describe a new protocol to allow inexpensive and low DNA input library prep for Illumina shotgun metagenome sequencing of microbiomes. This represents the most novel part of the paper, with the caveat that similar low input protocols have been described and used by others and were not mentioned here (see below). The remaining results were already known or highlight anticipated such as the limitations (but also the advantages in terms of low DNA input) of the Nextera protocol in terms of assembly quality. As such, the paper appears to be a biotech technique with no new biological or evolutionary insights into the microbiome of humans or other hosts/habitats. It appears that the authors have an interesting application of the technique to mice microbiomes but opted to not include the biological part of that work here. This reviewer thinks that overall the method is solid and well executed and possibly will be used by others in the field. However, the

reviewer would also like to see some direct comparisons or at least discussion to other similar miniaturized protocols used by others such as the JGI or the MIT groups (e.g., Paul Blainey and his team). The text is not always well-written or well-connected, and several ambiguities exist throughout (see also specific comments below). Some editorial editing will be need. Finally, mock community is the only way to do this type of project/work robustly and highly accurately, and it is not as labor intensive as the authors imply in the abstract (see also below). There was nowhere in the manuscript any data/results to suggest that what the authors did here could not have been done with a mock sample. For instance, the example provided in the middle of page 9 is a clear case where only mock samples can do this well. Even the long read Illumina technology proposed as an alternative is error-prone and costly that not many labs can afford it (plus Illumina has discontinued them due

probably to these limitations). I suggest revising the justification for the study to not sound so critical of mock samples and given a misleading view. The accession numbers for the sequencing data reported need to be made available before acceptance of the manuscript.

We very much appreciate the reviewer’s thoughtful comments on the manuscript. We have tried to address each of their concerns directly. In particular, we eliminated most of the text that could be read as critical of mock community-based benchmarks, and instead tried to present our approach simply as a potential alternative approach. We have also expanded the discussion of how our library protocol compares to other miniaturization approaches.

Minor-specific comments.

Ln 33-38, pg 5. I agree with the need, but the mock work is overly criticized in my view here. If one wants to do a real mock, one could just add DNA or cells of interest on a real reference stool sample. It is a bit more labor intensive but the alternative proposed here is not much cheaper either. I would not criticize mock work the way that the authors did and my suggestion to them is to lower the tone on this or completely remove these part.

We have removed or tempered the criticisms of mock community analysis as requested, both in the introduction and the discussion, and instead focused on presenting the long-read reference approach as an independent alternative of potential interest.

Ln 18-22, pg 7. I believe this result (and others mentioned elsewhere) is consistent with what Illumina has published. If not, please make it clear. Also, I am not sure the comparison per cost makes scientific sense; I think a comparison based on same number of sequences (total bp) makes more sense (and say what is the associated cost per bp).

We have added a clause to note that our findings are consistent with Illumina’s recommendations. Although we have seen this referenced in forums and blog posts, we have not been able to find explicit documentation of this phenomenon in the literature, and are hoping that readers will find the explicit comparison useful.

We respectfully disagree that cost-based comparisons should be disfavored in this context, as sequencing effort is often constrained by cost, and reducing total marginal cost of sample sequencing was one of the primary goals of our study.

Ln 34, pg 7. I am a bit surprised that the authors used one of the least performing binning algorithm out there. MaxBin, MetaBAT and other more recent algorithms have been shown to do better job that CONCOCT in general. See also the CAMI challenge papers for details. It seems this was used in terms of convenience with the Anvi'o pipeline and likely did not affect results much though. Some discussion of this topic would have been useful, especially in terms of instability in CONCOCT or how fine tuning of its parameters could affect the assembly of datasets from different library creation methods differently.

We agree that CONCOCT is not the optimal binner, and have made a parenthetical comment to make sure the reader is aware of superior alternatives, as well as the possibility of evaluating performance using the unbinned long-read assemblies directly.

Because we performed this step prior to the development and optimization of our miniaturized library prep protocols, CAMI results were not yet available.

Pg 8. Why there is an almost empty sheet on page 8? Formatting issues?

There indeed seems to have been some issue with formatting. We have tried to make sure that the document translated correctly in our revised version of the manuscript.

Ln 4-9, pg 9. TruSeqNano was compared for the 5 best assembled genomes; why NexteraXT on 40? Seems inconsistent. Also, I suspect that some of the results may be due to higher similarity of these library making methods to the long-read method. Can the authors prove that this is not the case here? This seems to be critical information that is missing.

Each technology was compared to the top 5 genome bins for each available sample; thus, NexteraXT and TruSeqNano were both compared for the 5 best assembled genomes from each of 8 samples (total 40).

We agree that it is possible that library chemistry similarities could explain some of the observed assembly performance, and have added a paragraph at the end of this section explicitly drawing attention to this possibility.

Ln25-50, pg 9. This analysis is much better done with a mock (known) genome for instance on page 9.

We agree that mock communities would have enabled a broader suite of statistical assessments.

Ln34-35. pg 10. This is interesting but I think it is better for a more specialized journal on biotech techniques. In any case, what is the cost for the required infrastructure e.g., for liquid handlers? This should be discussed for the estimate of 7$/sample to not be misleading.

We agree that this is an important consideration, and have added a sentence to this section to remind the reader that the capital costs can be considerable. We have also added a new paragraph to the discussion discussing these capital costs, and comparing our protocol to microfluidics-based miniaturized protocols. Because capital cost accounting varies so much between institutions, and the per-sample fixed costs will vary with the overall numbers of samples, we don’t think we can meaningfully provide a ‘total’ per-sample estimate.

ln 20, pg 12. "commonly yielded ≈ megabases". Is "≈" a typo or what?

We intended for this to be read as “commonly yielded approximately megabases of assembly”, but have removed the “≈” to increase clarity.

Ln 53. BinS (typo)

Thank you for catching this! We have updated bins to be plural.

Ln 24-25, pg 14. bins and MAGs are the same thing; yet, it is a bit confusing how MAGs were used here.

We agree, and as we only used MAG a few places, have replaced the acronym with either “bins” or spelled it out, as appropriate.

Ln 24, pg 15. rRNA GENE profiling. (gene is missing)

We have changed “16S rRNA” to “16S rRNA gene”

Reviewer #2: The paper is broadly concerned with developing production scale metagenomes for large sample sets. This is key important problem in contemporary microbiome research, and this paper make95125131s some new contributions. The authors first consider how to define reference genomes using multiple-single sample assemblies constructed using synthetic long read data, obtaining reference genomes from most abundant community members: an approach they describe as 'leaderboard metagenomics' (although this strikes as a piece of unnecessary jargon). The second part of the paper evaluates the use of high throughput library constructions in the relation to genome recoverability. The data and findings of the second half of the work are particularly valuable in moving toward to production scale genome-resolved metagenomics.

The concept developed here is roughly that a community is sampled that has variable abundance dynamics, so it is plausible that by using sufficiently large samples, one can take advantage of many observations to recover a particular member genome with accuracy, and so obtain a cohort of high quality genomes from the community. The approach is actually implicit in the dRep workflow from Banfield's group (Olm; PMID: 28742071), in which cognate bins from multiple binned metagenome assemblies are identified and the best instantiation retained as a 'dereplicated' reference, and this paper should be mentioned in the Discussion. The approach was also used in 2014 by Nielsen et al (which the authors cite) in a gene-centric partial genome recovery method described as 'canopies' (and which has not been developed much further to the best of my knowledge). As the authors already highlight, the issue is related to the use of co-assemblies, although the relative advantages and

disadvantages of co-assemblies versus multiple single sample assemblies are still being worked out.

We appreciate the reviewers thoughtful comments, and have tried to address each of their comments in the text. In particular, we have expanded the introduction to better contextualize our “leaderboard” approach within the existing literature, and expanded the discussion to draw more explicit attention to the caveats of trading sequencing depth for sample breadth.

Specific comments:

p5, line 13. "...can be used for mapping based analysis of less abundant species and strain variants within each sample". Yes, they can but there will be genomes which cannot be well recovered and that do not have conspecifics in the most abundant recovered set, which be missed by this approach. Ultimately it depends on how complex the community is in terms of number of species (richness), evenness (high evenness communities will be more difficult to analyse) and genetic/genomic diversity, and some mention should be made of these constraints in the Discussion; see also note below.

This is an important point, and we agree that the initial discussion did not adequately address it. We have added an additional paragraph to the discussion considering these caveats.

p5, line 53-54. "...apply to other highly accurate long read technologies as they emerge". Recent work on long read MAG analysis should be cited here (Arumugam, 2019; PMID:30992083 and Nicholls, 2019; PMID:31089679 and Somerville PMID:31238873).

We have added some citations for more recent accurate long-read sequencing approaches.

p9, paragraph 2, line 24. Does "mismatch" simply refer to the total number of observed alignment mismatches between the test assemblies the TSLR references?. I may have missed it, but as far as I can tell this is not explained anywhere.

p9 paragraph 2, line29. "....with lower coverage bins...capacity for internal error correction (Fig 2)." is very unclear. Please try and improve the clarity of this sentence.

Yes, this section refers to mismatches between the short-read assemblies and the TSLR references. We have rewritten these sentences to try to clarify.

p12, last paragraph. What is unclear here is the degree of redundancy in the set of bins recovered these analyses. Would recommend the authors apply the dRep workflow to determine the number of secondary clusters, which represents the actual number of dereplicated genomes present. Additionally, the total number of bins detected in low, medium and high quality levels (i.e. MINIMAG criteria of Bowers) should be reported.

This is an excellent point. We have added an analysis of the complete genome bin set using the dRep workflow as suggested, and included an additional figure.

p13-p15. Finally, there are aspects to the Discussion that need some balance. One impression is that this paper may implicitly convey to the non-expert reader is that it is only top most abundant taxa that are important, and secondly that communities will be variable enough over time that by using repeated surveys that individual member genomes will be able to be recovered across the a reasonably broad abundance range. Neither assumption is demonstrated to be the case in this analysis, and my concern is that this will get misinterpreted and mis-cited by naive or over-enthusiastic practitioners. I agree that given sufficient samples of the same community type the concept of "expand[ing]...organically" (p14, line26) is logical and compelling, however, this is may be feasible only for communities of limited complexity, and it remains unclear whether repeated observations, coupled with MAG analysis as performed here, we be able to resolve more elusive member genomes of complex

communities such as activated sludge or soil. In part this opinion also relates to my comment above about the unnecessary terminology of 'leaderboard metagenomics'.

We agree that more nuance needed to be conveyed here. As such, we have added a new paragraph to the discussion explicitly noting the caveats with respect to community complexity, as well as the inherent limitations with respect to always-rare genomes.

p13, line 32. As stated above, such long read analyses have already appeared in the literature, so they should be cited here (see note about p5, line 53-54)

As above, we have added citations to these emerging technologies.

p16. Methods, line 4. There needs to be a lot more detail provided here about these sample. What was the duration between sampling? Can the authors please list the details of the IRB approval as per standard practice. When were the additional samples referred to line 29 of the same page taken?

We have updated the methods with more specific sourcing information, including IRB approval numbers, for these samples. We must apologise for the mention on line 29 to the additional samples from the time series; although we have generated data from additional samples in this series, those data were not included in this paper.

p16, line 52."we modified SPAdes". Can you provide some more detail? Is this covered by the material in the subsequent sentence starting "In particular..."? Is this code available?

An earlier version of this manuscript was initially intended as a companion paper to the truSPAdes paper, and the minor changes necessary to allow these parameters were made to the truSPAdes branch of the assembler. We have updated the text to cite the appropriate publication, which contains links to the software.

p17, p41. "manually refined". Do the authors have any logs or outputs from anvi-refine to show the extent of these procedures?

We have retained all the original data from Anvi’o. We have added a footnote to the supplemental table indicating which TSLR bins had any manual intervention.

**Second round of review**

**Reviewer 1**

The authors provided a much improved version of the manuscript and addressed all my major concerns. I have only a few minor concerns remaining:

Line 26, pg 9: reflecT (missing the "t" at the end of the word)

Line 16-17, pg 10. I dont follow the underlying reasoning. Can the authors explain better? Since the comparison is based on non-reference material it could still show more similar results for methods with more similar chemistries, NO? In other words, how exactly the non-reference comparison sidesteps the issue of more similar chemistries is not clear.

Line 57, pg. 10. "…will be higher", by 2 fold, 10 fold more? some estimate would be useful for the readers, even if rough, or even if it is just the catalog price for the companies. I appreciate the response of the authors about differences in prices between labs and Institutions but this also applies to the library kits to some extent. Hence, their response here is not fully satisfactory. 

Line 18, pg 14. typo. please check.

**Reviewer 2**

No further comments, the authors have addressed my concerns.

**Authors Response**

**Point-by-point responses to the reviewers’ comments:**

We very much appreciate the additional effort by the reviewers and editors on this manuscript. We have made all the requested revisions. Please see below for specific responses to Reviewer 1's additional comments.

[Reviewer 1]

Line 26, pg 9: reflecT (missing the "t" at the end of the word)

RESPONSE: Fixed, thank you.

Line 16-17, pg 10. I dont follow the underlying reasoning. Can the authors explain better? Since the comparison is based on non-reference material it could still show more similar results for methods with more similar chemistries, NO? In other words, how exactly the non-reference comparison sidesteps the issue of more similar chemistries is not clear.

RESPONSE: We have amended this text to clarify that we're talking about overall performance, rather than purely about reference-based performance. In other words, contiguity is improved overall across sample types, and not just in comparison to a particular reference.

Line 57, pg. 10. "…will be higher", by 2 fold, 10 fold more? some estimate would be useful for the readers, even if rough, or even if it is just the catalog price for the companies. I appreciate the response of the authors about differences in prices between labs and Institutions but this also applies to the library kits to some extent. Hence, their response here is not fully satisfactory.

RESPONSE: The consumables estimate is using catalogue prices, not any sort of special discounts, and is thus not likely to be dramatically different across institutions. We have amended the text to clarify. Capital equipment depreciation, however, is handled very differently between institutions, and can't be expressed on a per-sample basis without some estimate of the total quantity of samples processed over the operating life of the instrument.

Line 18, pg 14. typo. please check.

RESPONSE: We weren't sure exactly what the problem with this line was, so we have modified it to be a little more grammatically unambiguous.
